# A novel nonosteocytic regulatory mechanism of bone modeling

**DOI:** 10.1371/journal.pbio.3000140

**Published:** 2019-02-01

**Authors:** Lior Ofer, Mason N. Dean, Paul Zaslansky, Shiri Kult, Yulia Shwartz, Janna Zaretsky, Shelley Griess-Fishheimer, Efrat Monsonego-Ornan, Elazar Zelzer, Ron Shahar

**Affiliations:** 1 Koret School of Veterinary Medicine, The Robert H. Smith Faculty of Agriculture, Food and Environmental Sciences, The Hebrew University of Jerusalem, Rehovot, Israel; 2 Department of Biomaterials, Max Planck Institute of Colloids & Interfaces, Potsdam, Germany; 3 Department for Restorative and Preventive Dentistry, Charité-Universitaetsmedizin Berlin, Berlin, Germany; 4 Department of Molecular Genetics, Weizmann Institute of Science, Rehovot, Israel; 5 Department of Stem Cell and Regenerative Biology, Harvard, Cambridge, Massachusetts, United States of America; 6 Institute of Biochemistry and Nutrition, The Robert H. Smith Faculty of Agriculture, Food and Environmental Sciences, The Hebrew University of Jerusalem, Rehovot, Israel; University of Pennsylvania School of Medicine, UNITED STATES

## Abstract

Osteocytes, cells forming an elaborate network within the bones of most vertebrate taxa, are thought to be the master regulators of bone modeling, a process of coordinated, local bone-tissue deposition and removal that keeps bone strains at safe levels throughout life. Neoteleost fish, however, lack osteocytes and yet are known to be capable of bone modeling, although no osteocyte-independent modeling regulatory mechanism has so far been described. Here, we characterize a novel, to our knowledge, bone-modeling regulatory mechanism in a fish species (medaka), showing that although lacking osteocytes (i.e., internal mechanosensors), when loaded, medaka bones model in mechanically directed ways, successfully reducing high tissue strains. We establish that as in mammals, modeling in medaka is regulated by the *SOST* gene, demonstrating a mechanistic link between skeletal loading, *SOST* down-regulation, and intense bone deposition. However, whereas mammalian *SOST* is expressed almost exclusively by osteocytes, in both medaka and zebrafish (a species with osteocytic bones), *SOST* is expressed by a variety of nonosteocytic cells, none of which reside within the bone bulk. These findings argue that in fishes (and perhaps other vertebrates), nonosteocytic skeletal cells are both sensors and responders, shouldering duties believed exclusive to osteocytes. This previously unrecognized, *SOST*-dependent, osteocyte-independent mechanism challenges current paradigms of osteocyte exclusivity in bone-modeling regulation, suggesting the existence of multivariate feedback networks in bone modeling—perhaps also in mammalian bones—and thus arguing for the possibility of untapped potential for cell targets in bone therapeutics.

## Abstract 2

Bone’s ability to change its morphology in response to load is widely attributed to osteocytes. A study of fish shows that bone can respond to load even in the absence of osteocytes, using a molecular mechanism that is conserved across vertebrates, albeit with different cellular effectors.

## Author summary

Bone is a “smart” tissue, able to sense loads within its bulk and change its morphology when needed by a process named bone modeling. This process is carried out by bone-depositing cells (osteoblasts) and bone-resorbing cells (osteoclasts) and is regulated by osteocytes—cells that reside in small cavities within the bone tissue. Osteocytes are considered to function as mechanosensors, detecting areas of high loads that require modeling, and master regulators of osteoblasts and osteoclasts. Curiously, evolutionarily advanced fish do not have osteocytes in their bones, although more basal fish and all other bony vertebrates have them. In this paper, we show how the bones of advanced fish can respond to load in a mechanically efficient way despite the absence of osteocytes. We describe the molecular mechanism, which we found to be the same as in all other vertebrates; however, we show that the cellular effectors are different. The protein sclerostin, which is produced by osteocytes in mammals and is a potent suppressor of bone building by osteoblasts, is produced by a variety of nonosteocytic cells in medaka and zebrafish, and nonosteocytic skeletal cells serve as sensors and responders in these species. These results challenge current paradigms of osteocyte exclusivity in the regulation of bone modeling.

## Introduction

Fish bone is comprised of the same material building blocks as mammalian bone (mineral, water, collagen, and other proteins) [1]. As in mammalian bone, fish bone also possesses both bone-depositing (osteoblast) and bone-resorbing (osteoclast) cells, the building and wrecking crews of the bone-modeling response [2,3]. It is the bone-modeling process—the addition or removal of bone tissue to or from bone surfaces—that grants bone the ability to respond adaptively to changing loads [4–6]. However, the bones of most advanced fishes (neoteleosts) completely lack osteocytes, which are present in huge numbers and constitute over 90% of all cells in the bones of all other vertebrate taxa, including basal fishes [1,3,7–9]. Osteocytes are considered the architects of modeling, directing osteoblast and osteoclast action [4,10]. Although the mechanosensing and regulatory functions of osteocytes in bone, particularly with regard to modeling, have not been confirmed incontrovertibly in vivo [11–13], the density and connectivity of the osteocyte network makes these putative roles very likely [14].

Mammalian paradigms argue therefore that neoteleost fish should be at a functional disadvantage because of the absence of osteocytes, unable to adapt their bones to changing loads. However, several studies demonstrated that anosteocytic fish bones do respond to their mechanical environment by modeling (e.g., [3,15–17], particularly in response to swimming [18–20]), though the mechanisms and cellular effectors of this response remain unknown. Anosteocytic fish bone, a natural osteocyte knock-out, therefore offers a unique model for studying the regulatory mechanism of bone modeling and for investigating the widely accepted primacy of osteocytes in bone biology.

## Results

### Modeling in response to swimming

**Fig 1:**
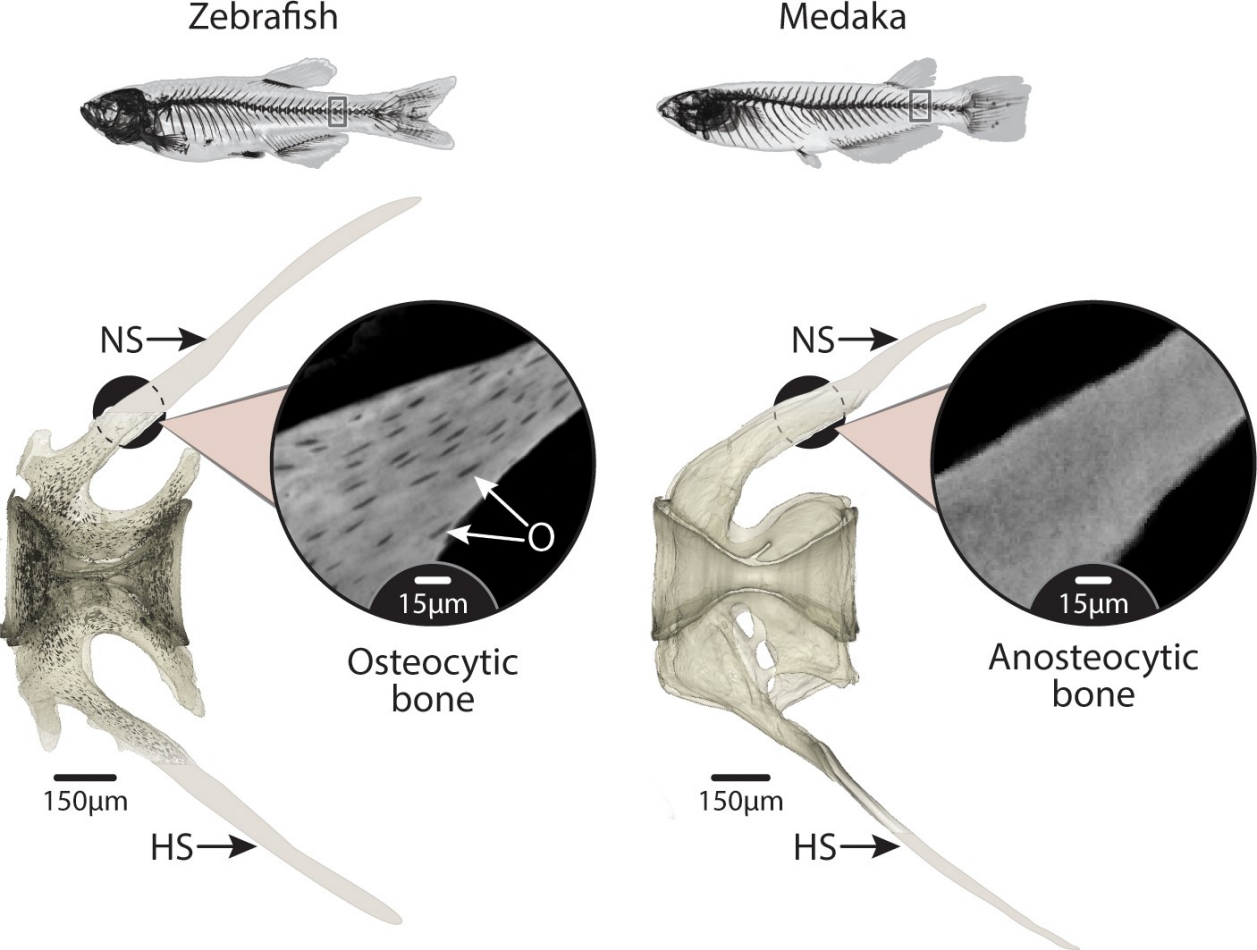
Anosteocytic vertebra of medaka and osteocytic vertebra of zebrafish. High-resolution tomography of caudal vertebrae of zebrafish (left) and medaka (right). O, colored in black in the zebrafish scan, show the ubiquity of cells residing in the bone material of zebrafish while being completely absent from medaka bone material. Note that the distal parts of the NS and HS were cropped in the original scans and are only drawn here for reference; therefore, these parts do not contain lacunae in the zebrafish rendering. Inset images show unsegmented tomography slices at a higher magnification. The vertebrae of both species are hourglass shaped along the cranio–caudal axis (see 3D representation in Fig 3A), with NS and HS extending caudally from the vertebral body. HS, hemal spine; NS, neural spine; O, osteocytic lacunae.

In order to critically examine the role osteocytes play in bone modeling, we compared the skeletal response to loading in medaka and zebrafish (Fig 1), two common laboratory fish species differing in their bone-tissue type—being anosteocytic and osteocytic, respectively—but otherwise similar in size, ecology, and swimming mode. Because fish are almost neutrally buoyant, their skeletons are loaded primarily by muscle forces. We regulated skeletal loading conditions using a custom-built swim-training system, requiring fish to swim against a semilaminar water current of tightly controlled velocity (videos of swim training are shown in S1 Video). Strenuous swim training increases the load applied by the powerful axial paravertebral muscles and tendons, which attach to the vertebrae and are responsible for the oscillatory movements of swimming [18,19], providing a tractable framework for examination of the modeling response due to exercise (S1 Fig). To visualize new bone formation, we injected individuals intraperitoneally with calcium-binding fluorochromes (alizarin red at time zero and calcein green after 6 weeks of strenuous swimming), then compared the extent and spatiotemporal dynamics of new bone formation in caudal vertebrae between experimental (swim-trained) and control (non-swim-trained) fishes and between the osteocytic and anosteocytic species.

**Fig 2:**
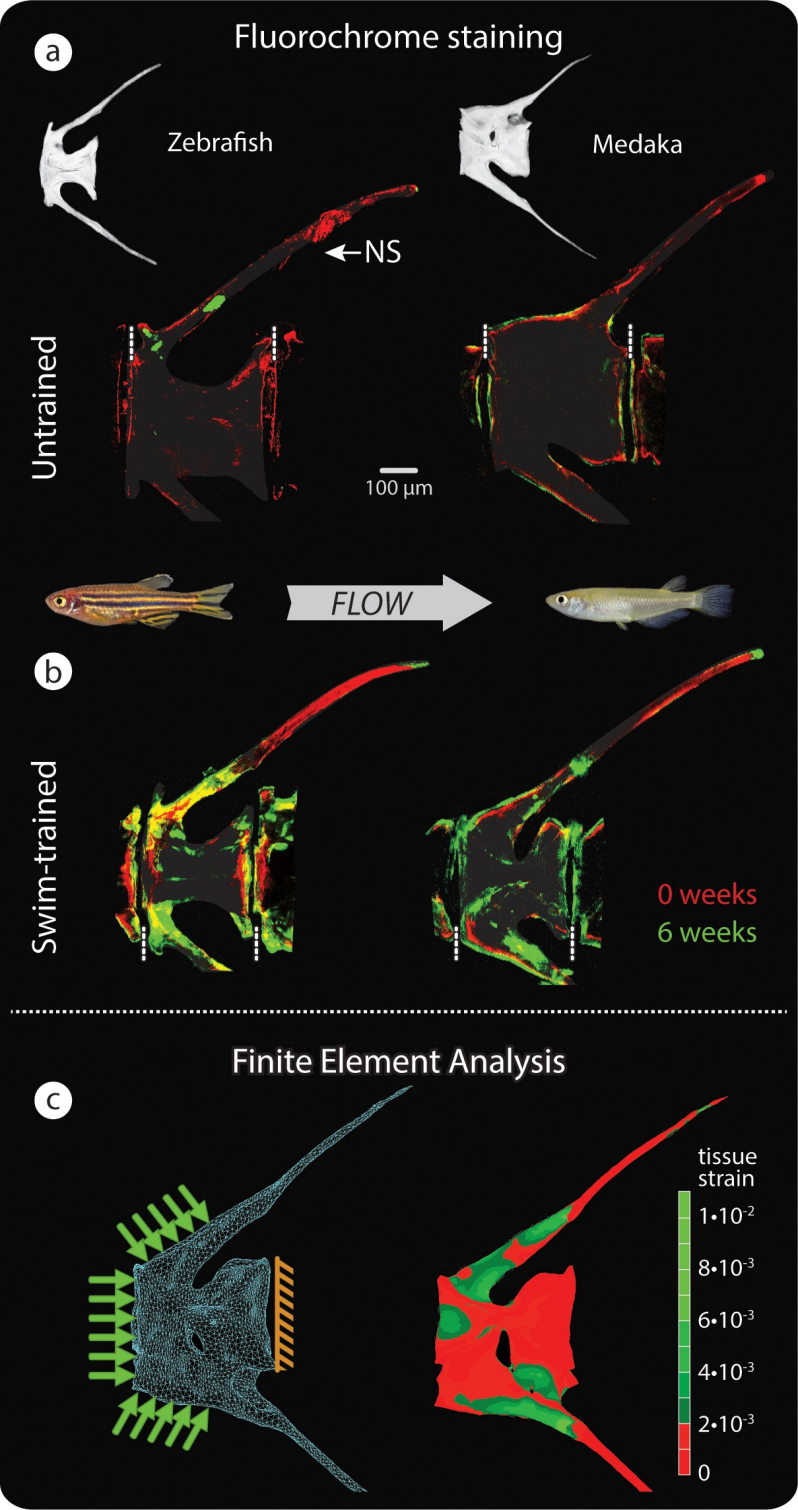
Vertebral bone formation in response to load. (a–b) Bone formation detected by sequential intraperitoneal fluorochrome injections in untrained (a) and swim-trained (b) zebrafish (left, osteocytic) and medaka (right, anosteocytic), each of the 4 groups comprising *n* = 4 fish. Alizarin red was injected at *t* = 0 weeks, and calcein green was injected at *t* = 6 weeks of swim training. Dashed, vertical white lines mark the border between vertebrae. (c) FE model (left) and FEA results (right), showing von Mises strains in a loaded medaka vertebra. Note similarity of peak strains predicted by FEA in (c) and regions of intense bone formation in response to load, indicated by fluorochrome double labeling (b). The distal HSs are cropped in fluorochrome and FE renderings. FE, finite element; FEA, finite element analysis; HS, hemal spine; NS, neural spine.

The current osteocyte-centric dogma of bone biology argues that modeling in the osteocyte-rich skeleton of zebrafish should be far more efficient and spatially targeted, with osteocytes sensing loads locally throughout the bulk of the tissue and directing osteoblasts to build new tissue in appropriate locations experiencing maximal strains [21,22]. In contrast, the anosteocytic bones of medaka should lack the ability to mechanosense and regulate modeling, and therefore, modeled bone should be randomly distributed or at least not correlated with tissue strains. However, our results indicate that the modeling behavior is strikingly similar in the 2 species. In both medaka and zebrafish, we observed minimal bone formation in the vertebrae of untrained (nonexercised) individuals (Fig 2A). In contrast, swim-trained fish of both species exhibited new bone formation of similar extent and spatial distribution (Fig 2B). Our nanoindentation results show that in both zebrafish and medaka, the stiffness (Young’s modulus) of the vertebral bone tissue is not significantly different between swim-trained and untrained fish (S2 Fig). The locations of new growth in swim-trained fish correspond closely with regions where high loads are expected: regions of muscle attachment (e.g., along the neural arch and proximal spine) and of articulation with adjacent vertebrae (e.g., the cranial and caudal edges of the vertebral body bordering the intervertebral joint space).

### FEA predicts modeling response to swimming

In order to assess the correlation between regions where high loads are expected and locations of peak strains, we performed finite element analysis (FEA) on a 3D computer model that simulated a loaded medaka vertebra. The model incorporated detailed structural and material data collected by high-resolution tomography scans and mechanical testing and enabled calculation of the 3D strain distribution in a swim-trained medaka vertebra. The results of these simulations confirm that new tissue deposition, as demonstrated by fluorochromes, is prominent in regions predicted by FEA to experience peak strains (the presumed stimulus for modeling [21,22]; see Fig 2B and 2C).

**Fig 3:**
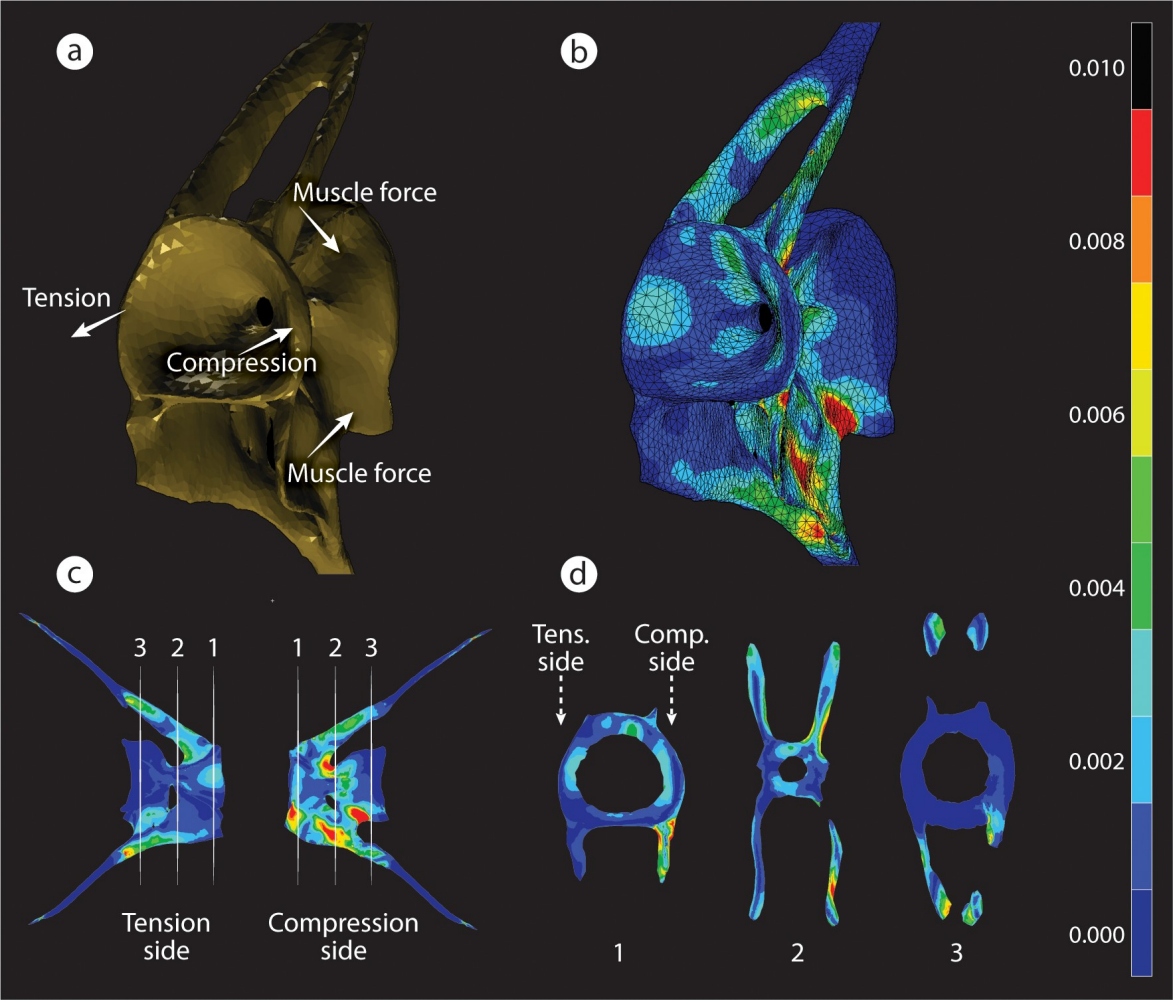
FEA of strain distribution in a loaded medaka vertebra. (a) A simplified loading graphic of the forces applied to the model. Lateral bending of the vertebral column during oscillatory swimming creates forces on the flat articular surfaces of adjacent centra, F Comp. on one side and F tens. on the opposite side. F Muscs acting on fish vertebrae are rather difficult to model because of the high complexity of the musculature as shown in S1 Fig and are represented here in a simplified manner. (b) 3D representation of von Mises strains in the vertebra. (c) Strain distribution in 2 contralateral views of a loaded vertebra. Locations of several transverse sections are marked by white dashed lines and are shown in (d). (d) Strain distribution in the 3 numbered 2D transverse sections shown in (c). Peak strains occur primarily near the external surfaces of the VB. Strain magnitudes (no units) for all images are shown in the color bar on the right. F Comp., compression force; FEA, finite element analysis; F Musc, muscle force; F Tens., tension force; VB, vertebral bone.

Vertebral modeling in response to swim training, coupled with the close agreement between strain-distribution predictions and tissue-deposition patterns seen in our experimental data, argues that bone modeling in medaka vertebrae is not random, despite the lack of osteocytes, but rather closely correlated to the strain environment arising in the bone during swimming. More significantly, this mechanically relevant tissue response confirms that modeling is possible and directable even without the presence of numerous mechanosensors located within the bulk of the bone tissue. This would suggest that modeling in anosteocytic medaka either does not require internal strain information and relies only on sensors on the external surfaces of the bone or uses external sensors to read internal strains (perhaps via the array of the densely packed, hypomineralized fibers that perforate fish bone [23]). Detailed results of FEAs furthermore indicate that peak strains occur primarily near the external surfaces of the vertebral bone (Fig 3), supporting the potential efficiency of surface sensors.

### *SOST* expression in anosteocytic bone

In mammals, one of the major regulators of bone’s response to mechanical loads is the *SOST* gene, which is expressed almost exclusively by osteocytes and encodes the protein sclerostin, a potent suppressor of bone building by osteoblasts [24–26]. During skeletal loading in mammals, *SOST* expression by osteocytes is down-regulated, releasing osteoblast inhibition [13,25] and promoting local bone deposition. Therefore, modulation of *SOST* expression by osteocytes is considered an important bellwether for bone modeling in mammals. The absolute lack of osteocytes in medaka bones guarantees that their bone-modeling regulatory pathways (cellular, molecular, or both) differ from those of mammals. The cellular and molecular pathways of modeling regulation in fishes, however, are unknown, hampering a broader and phylogenetic perspective on the physiology of bone. Demonstration of *SOST* expression in medaka, e.g., would indicate *SOST*-mediated modeling regulation is possible but by a nonosteocytic cell, whereas lack of *SOST* expression would suggest other molecular regulation pathways (i.e., via other genes or gene products).

**Fig 4:**
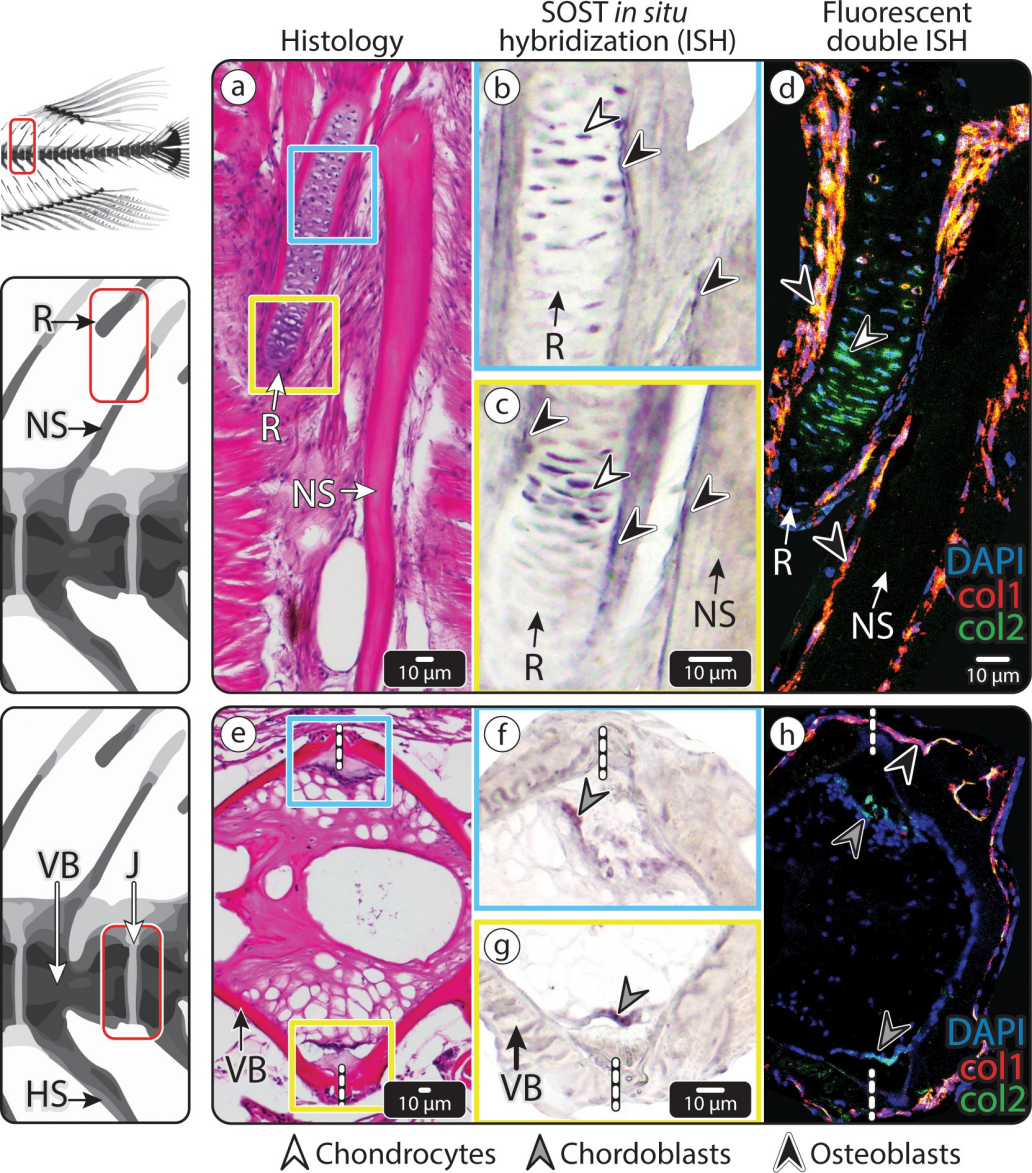
*SOST* expression in medaka vertebra and dorsal fin R. (a–d) An NS of medaka vertebra and adjacent fin R. (a) HE-stained section. The blue and yellow squares mark 2 regions in the dorsal fin R, shown in (b) and (c), respectively. (b–c) ISH showing *SOST* expression in the cartilaginous core of the dorsal fin R (white arrowheads) and surface osteoblasts of the fin R and NS (black arrowheads). (d) Double fluorescent ISH, showing both *Col1a1* (red) and *Col2a1* (green) expression in the *SOST*-positive osteoblasts (black arrowheads) of the NS and fin R and *Col2a1* in the *SOST*-positive chondrocytes (white arrowhead) in the cartilaginous core of the fin R. (e–h) The IVR in the caudal vertebral column of medaka, (e) HE-stained section. The blue and yellow squares mark the 2 regions of the intravertebral region shown in (f) and (g), respectively. (f–g) ISH showing *SOST* expression in chordoblasts (gray arrowheads), identified by their distinct morphology and location. (h) Double fluorescent ISH in the IVR, showing *Col1a1* expression (red) in osteoblasts (black arrowhead) in the external region of the IVR and *Col2a1* expression (green) in *SOST*-positive chordoblasts (gray arrowhead) in the internal region of the IVR (scale bar same as in e). Dashed, vertical white lines mark the border between vertebrae. Results for individual stains (e.g., DAPI, *SOST*, col1, col2) are shown in S4 Fig. *Col1a1*, collagen type I alpha 1; *Col2a1*, collagen type II alpha 1; HE, hematoxylin–eosin; ISH, in situ hybridization; IVR, intervertebral region; J, joint; NS, neural spine; R, radial; VB, vertebral bone.

We found, using in situ hybridization (ISH) on medaka vertebrae sections, that *SOST* was indeed expressed in the vertebrae of untrained (control) medaka, with particularly high expression levels along the margins of the intervertebral regions (IVRs) and neural spines (NSs), as well as within the core of the adjacent fin radials (Rs; the elements that support the dorsal fin rays and consist of a cartilaginous core surrounded by a bony collar) (Fig 4B, 4C, 4F and 4G). We established that *SOST* was expressed by a diversity of cell types in these regions, using histological staining (hematoxylin–eosin [HE], Fig 4A and 4E) and double fluorescent ISH for an osteoblast marker (collagen type I alpha 1 [*Col1a1*]) and a chondrocyte marker (collagen type II alpha 1 [*Col2a1*]) on serial sections (Fig 4D and 4H). *SOST*-expressing cells in the core of fin Rs were chondrocytes, as confirmed by their morphology and *Col2a1* expression (Fig 4B and 4C). The *SOST*-positive cells on the surfaces of both the spine and the fin R, however, were osteoblasts, as evidenced by their expression of *Col1a1* or both *Col1a1* and *Col2a1*, as well as their position, morphology, and gene expression patterns (Fig 4D) [27]. *SOST*-expressing cells in the IVR were identified as chordoblasts, based on their expression of *Col2a1*, their location, and their morphology [28,29] (Fig 4H).

**Fig 5:**
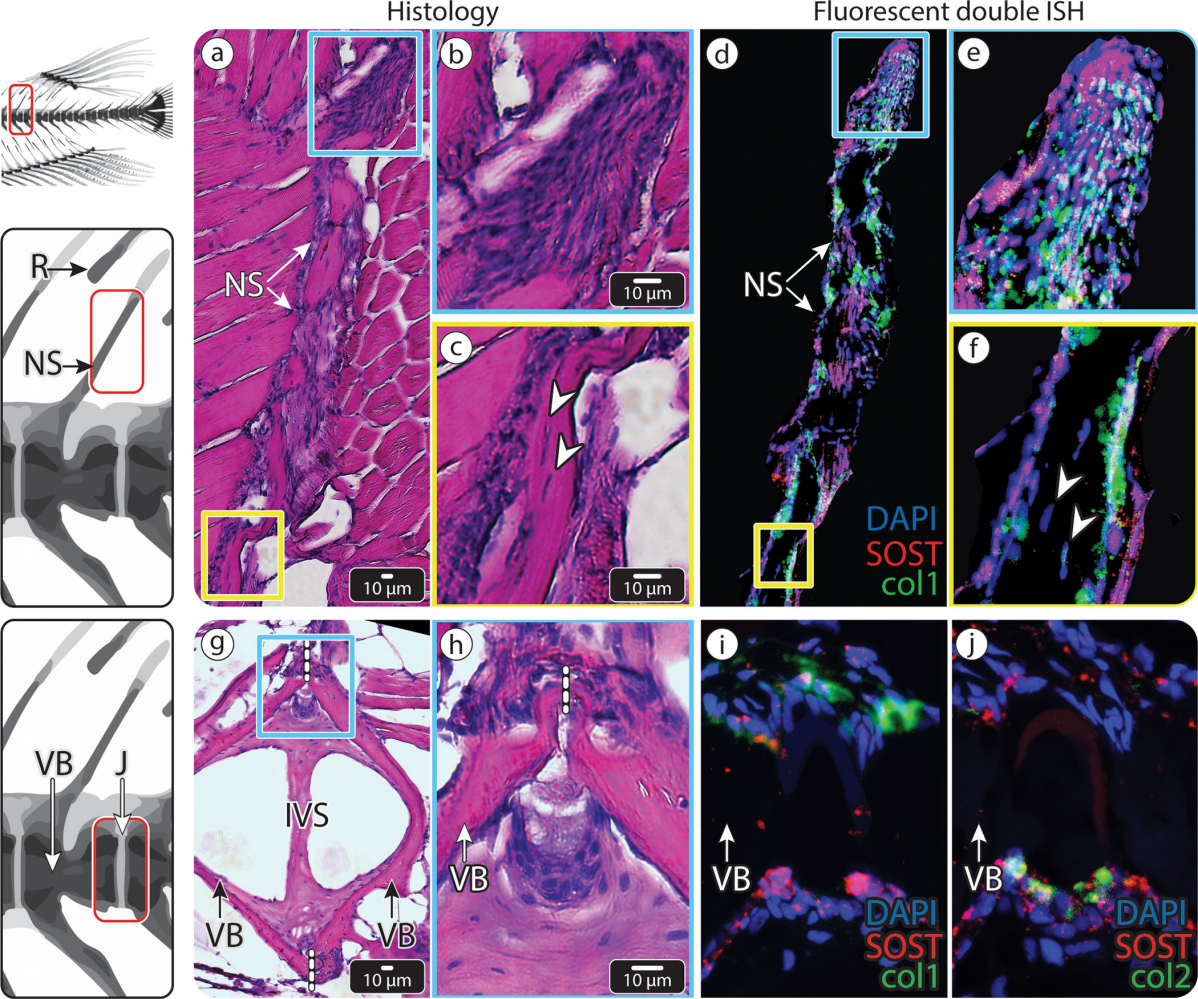
*SOST* expression in zebrafish vertebra. (a–f) An NS of zebrafish vertebra. (a) HE-stained section. The blue and yellow squares mark distal and proximal regions within the NS, respectively, shown at higher magnification in (b) and (c), respectively. (b) Higher magnification of the tip of the spine. (c) Higher magnification of the spine, showing osteocytes residing inside the bone material (white arrowheads). (d) Double fluorescent ISH showing both *Col1a1* (green) and *SOST* (red) expression in surface cells of the NS, indicating they are *SOST*-positive osteoblasts. The blue and yellow squares correspond to the regions (b) and (c) in (a) and to (e) and (f). (e) The tip of the spine contains numerous *SOST*-positive osteoblasts and seems to serve as an active growth region. (f) Osteocytes (white arrowheads) do not appear to express *SOST*. (g–j) The IVR in the caudal vertebral column of zebrafish, (g) HE-stained sagittal section. Dashed white lines mark the border between one vertebra to another. The blue square marks the intravertebral region shown in (h–j) at higher magnification. (h) Magnification of the IVR, showing the J between 2 adjacent VBs and their distinct cell populations. (i) Double fluorescent ISH (*SOST* and *Col1a1*) identifies the upper cell population in (h) as osteoblasts since they express *Col1a1*a (green) and shows both the upper and lower cell populations to express *SOST*. (j) Double fluorescent ISH (*SOST* and *Col2a1*) shows the lower population of cells to be chordoblasts, based on their distinctive morphology location and expression of *Col2a1* (green). These cells also express *SOST* (red). Scale bars apply to all images of comparable regions (i.e., b and e; c and f; h, i, and j). Results for individual stains (e.g. DAPI, *SOST*, col1, col2) are shown in S5 Fig. *Col1a1*, collagen type I alpha 1; *Col2a1*, collagen type II alpha 1; HE, hematoxylin–eosin; ISH, in situ hybridization; IVR, intervertebral region; IVS, intervertebral space; J, joint; NS, neural spine; R, radial; VB, vertebral bone.

Surprisingly, double fluorescent ISH performed on sections of the osteocytic vertebrae of zebrafish showed results similar to those seen in the anosteocytic vertebrae of medaka, with *SOST* expression seen primarily in chordoblasts and osteoblasts and weakly, if at all, in osteocytes (Fig 5).

### Modulation of *SOST* expression with exercise

**Fig 6:**
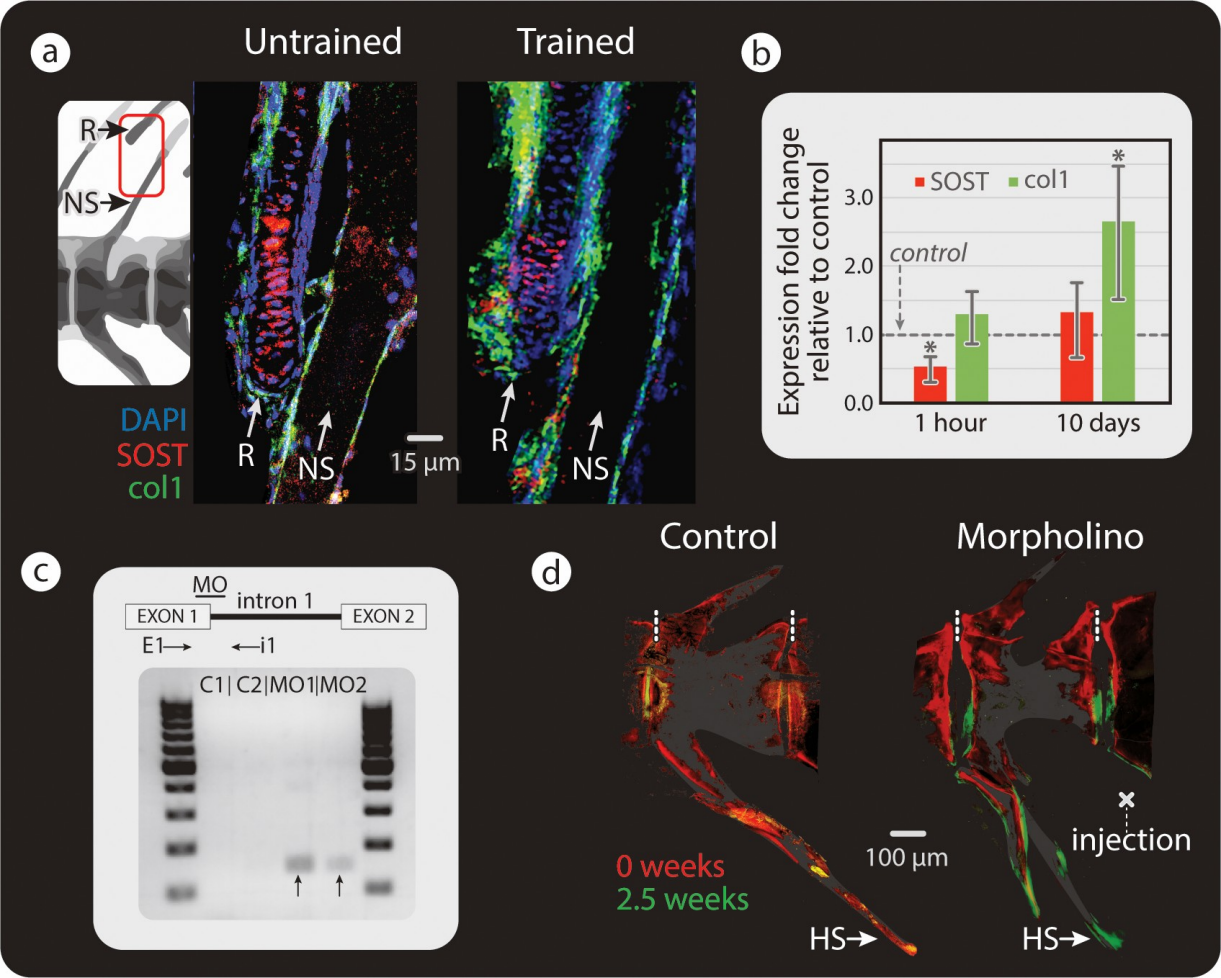
Mechanical load and *SOST* down-regulation cause increased bone formation. (a) Double fluorescent ISH, showing *SOST* (red) and *Col1a1* (green) in the spine and adjacent R of untrained (left) and trained (right) medaka (*n* = 3 for each group). In trained medaka, *SOST* expression appears down-regulated (as inferred from changes in staining intensity), while osteoblast recruitment is increased. (b) RT-qPCR results of *SOST* and *Col1a1* expression after 1 hour (*SOST*: swim trained *n* = 5, untrained *n* = 7 and *Col1a1*: swim trained *n* = 4, untrained *n* = 6) and 10 days of swim training of medaka (for both *SOST* and *Col1a1*: swim trained *n* = 5, untrained *n* = 4), (compared to levels in untrained medaka: gray, horizontal “control” line). Results were normalized to the rlp-7 housekeeping gene. Asterisks indicate a significant difference (*p* < 0.05) between trained and untrained (control) fish. Original raw data can be found in S1 Data. (c) Top: illustration of splice site blocking MO-binding site and primers used to detect morphant transcripts (E1, i1). Bottom: RT-PCR of medaka injected with standard control (C1, C2) and with *SOST* MO (MO1, MO2). Bands indicative of *SOST* splice blocking appeared in *SOST* MO-injected samples (black arrows) and not in the standard control samples. (d) Bone-formation dynamics marked by fluorochromes (red: alizarin red, injected at *t* = 0 weeks; green: calcein green, injected at *t* = 2.5 weeks) in vertebra of medaka injected with standard control MO (left) and *SOST* MO (right). Dashed, vertical white lines mark the border between vertebrae. NSs are cropped in fluorescence images in (d). *Col1a1*, collagen type I alpha 1; E1, exon-spanning forward primer; HS, hemal spine; ISH, in situ hybridization; i1, intron-spanning reverse primer; MO, vivo-morpholino; NS, neural spine; PCR, polymerase chain reaction; R, radial; RT-PCR, reverse transcription PCR; RT-qPCR, real-time quantitative PCR.

The finding that *SOST* is expressed in anosteocytic bone by osteoblasts, chondrocytes, and chordoblasts is intriguing since it raises the possibility that these cells may be involved in modeling regulation. Evidence of *SOST* expression, however, does not necessarily mean that *SOST* expression levels are dependent on loading. We therefore compared *SOST* expression levels in untrained medaka with those of medaka swim trained for 1 hour and for 10 days. Qualitative comparisons of double fluorescent ISH of *SOST* and *Col1a1* (Fig 6A) showed a decrease in *SOST* signal intensity after short-term swim training, suggesting that as in mammals, loading results in decreased *SOST* expression. These results were verified by real-time quantitative polymerase chain reaction (RT-qPCR), showing *SOST* expression levels decreased significantly (implying reduced inhibition of osteoblast activity) after 1 hour of swim training compared to those in the vertebral column of untrained fish (Fig 6B). Concomitantly, *Col1a1* expression began to increase, though not to a level significantly different from controls, implying the beginning of osteoblast recruitment. After 10 days of swim training, *SOST* expression had returned to baseline, whereas *Col1a1* expression had significantly increased (Fig 6B). All original raw data obtained by RT-qPCR analysis can be found in S1 Data. The asynchrony of *SOST* expression and *Col1a1* production suggests that bone loading promotes rapid down-regulation of *SOST*, but that the effect of inhibition release on osteoblast proliferation takes time to take effect. The increase in *SOST* expression once osteoblasts have reached peak stimulation (manifested by massively increased expression levels of *Col1a1*) points to a negative feedback mechanism in medaka skeletons, probably a sclerostin-mediated defense against excessive bone deposition, as employed by mammalian osteocytes embedded in newly modeled or remodeled bone [30,31]. These results point to *SOST* expression levels serving the same regulatory function of the bone-modeling process in anosteocytic fish bone as in osteocytic mammalian bone despite the different cellular origin of *SOST*.

### *SOST* down-regulation and bone deposition

To further verify that *SOST* expression levels in medaka are directly linked to bone formation, we injected fish with a custom-designed vivo-morpholino (MO) construct once every 3 days for 2 and a half weeks. The morpholino was designed to cause splice modification of the *SOST* gene (confirmed by gel electrophoresis; see Fig 6C), thereby mimicking the physiologic result of skeletal loading by effecting a knockdown of *SOST* expression and a decrease in sclerostin levels, albeit without actual skeletal loading. These fish were therefore not swim trained in order to ensure that any observed changes in bone deposition were the direct result of *SOST* down-regulation and not some other byproduct of exercise. Injections were performed close to the ventral aspect of a caudal vertebra (Fig 6D), and bone growth was visualized with fluorochrome staining at the beginning and end of the experimental period. Intense bone formation was indeed observed close to the injection site, where *SOST* expression had been locally knocked down (Fig 6D). This is in contrast to the lack of new growth at the same vertebral site in control animals, medaka injected with standard control (mismatch) morpholino. Vertebral regions remote from the site of injection (e.g., the dorsal aspect of the injected vertebra) also acted like controls, showing no increase in bone formation.

## Discussion

**Fig 7:**
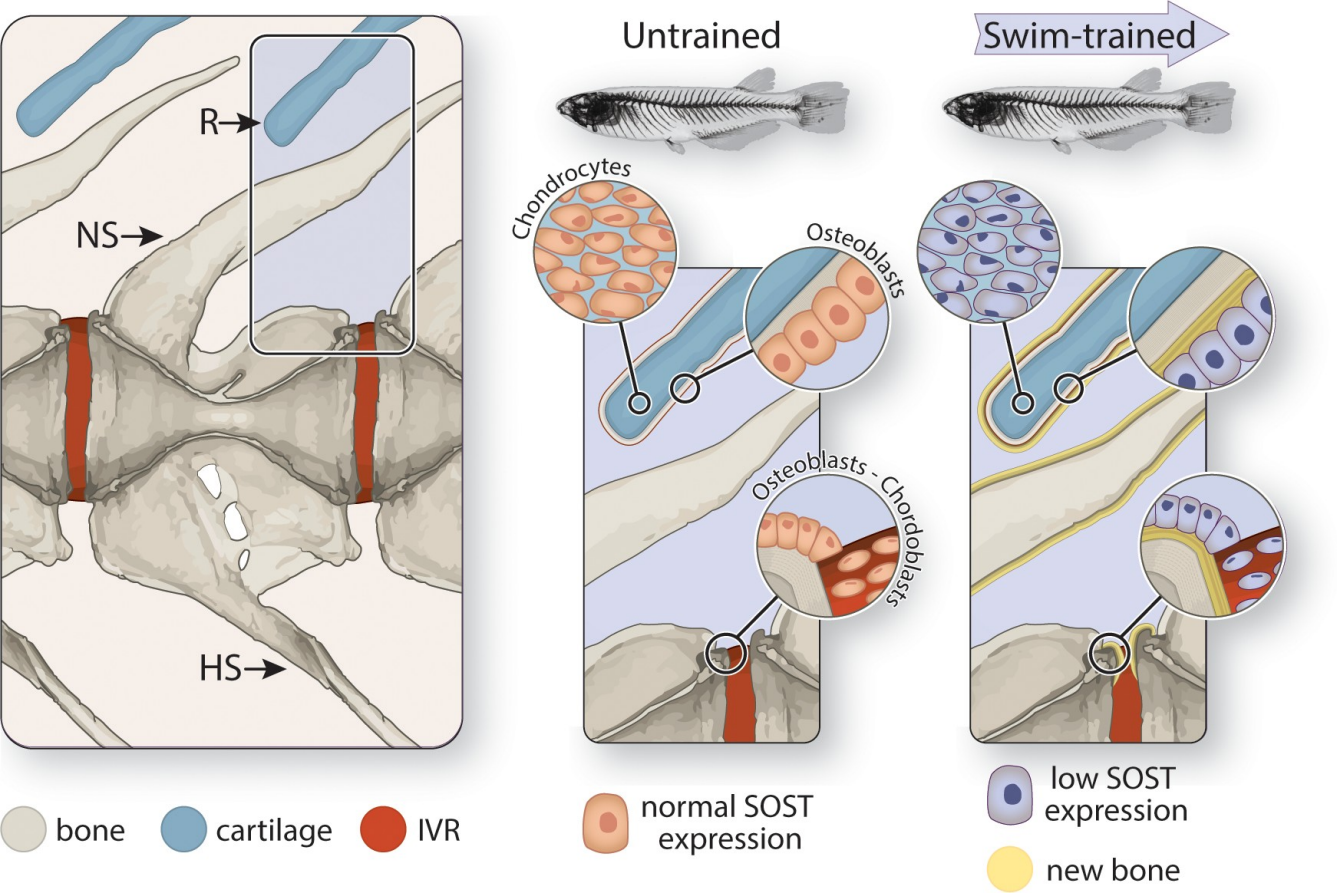
Schematic description of the proposed mechanism of osteocyte-independent modeling regulation in medaka vertebrae. (a) Medaka vertebra, showing the IVR, NS, HS, and R. (b) In the untrained vertebra, the chondrocytes, osteoblasts, and chordoblasts express normal levels of *SOST*, which maintains normal bone-deposition rate. (c) During swim training, the vertebra experiences increased strains, sensed by the osteoblasts, chondrocytes, and chordoblasts, leading to decreased *SOST* expression, which reduces osteoblast inhibition and results in increased bone-deposition rates. Similar, nonosteocytic *SOST*-mediated bone-modeling regulation was observed in the osteocytic skeletons of zebrafish, with some weak *SOST* expression by osteocytes as well. HS, hemal spine; IVR, intervertebral region; NS, neural spine; R, radial.

Our results provide convincing evidence for the regulatory role of *SOST* in modeling of the anosteocytic skeleton by showing the association between decreasing *SOST*-expression levels and increased bone deposition in the medaka skeleton (Fig 6). In this study, we present evidence for the existence of a successful osteocyte-independent mechanism that does not require a dense network of interconnected sensors in the bulk of the material but relies on regulation by surface cells that express *SOST* (Fig 7). In this way, our findings illustrate that the dogma of osteocyte exclusivity in bone-modeling regulation does not apply to all vertebrates, in the process also raising doubts as to its accuracy among mammals.

By evincing a shared bone-building response mechanism in osteocytic and anosteocytic bone types, our observations provide experimental validation for recent speculations that vertebrate skeletal mechanobiology is less osteocyte-centric than currently believed [11,12,32]. Sporadic reports have noted *SOST* expression by several nonosteocytic cells in mammals—by hypertrophic and osteoarthritic chondrocytes [33,34], osteoblast-like osteosarcoma cells [35,36], and even in normal osteoblasts, albeit at low levels [25,37]; however, these observations were not linked to bone-modeling regulation. Mammalian chondrocytes and osteoblasts are both mechanoreceptive, although osteoblasts were somewhat less sensitive to strain and fluid shear than osteocytes [38,39]. Results of our FEAs indicate that peak strains occur primarily near the external surfaces of the vertebral bone (Fig 3). This finding lends credence to the possibility that mechanosensors like osteoblasts, located only on bone surfaces, can provide information that is sufficient to orchestrate a “mechanically smart” response to loading despite the absence of osteocytes and therefore of strain data from within the bone matrix.

The involvement of *SOST* in osteogenesis regulation in both fishes and mammals and in bones with and without osteocytes indicates a fundamental conservation across vertebrates at the level of the molecular machinery—but not the cellular agents—controlling the adaptation of bone to mechanical loads. Our findings establish a mechanistic link between skeletal loading, local modulation of *SOST* expression, and bone modeling in both anosteocytic and osteocytic fishes (Fig 7). The demonstrated multifunctionality of fish osteoblasts, chondrocytes, and chordoblasts—sensing loads and regulating tissue deposition via *SOST*, even when osteocytes are present—provides the first experimental support for speculations that nonosteocytic cells can assume some duties of mammalian osteocytes [3,12,17,40].

The existence of an effective, osteocyte-independent modeling process in fishes raises intriguing possibilities with regard to the evolution of bone modeling, the roles played by various mesenchymal cells, and how these roles may have changed. On the one hand, fish and mammals may simply have different cellular effectors of bone modeling, even if they share a dependence on *SOST*. It is difficult to determine which cellular agents mediated bone modeling in stem vertebrates. However, given the apparent lack of reliance on osteocytes in fishes, the “mammal-like” osteocyte-dependent mechanism of bone modeling may have arisen or increased in prominence during vertebrate evolution, perhaps in the water-to-land transition, when gravitational loads on the skeletal changed drastically [41]. Similarly, the “fish-like” osteocyte-independent mechanism of bone modeling may have been lost. Alternatively, such a mechanism may exist in mammals, perhaps acting synergistically with osteocytic regulation but unrecognized until now because of the research emphasis on osteocytes.

Osteocytes are derived from osteoblasts, which in turn arise from pluripotent, mesenchymal stem cells capable of becoming osteoblasts or chondroblasts. Our illustration of *SOST* expression by osteoblasts in the bones of both osteocytic and anosteocytic fishes suggests an ancient association of *SOST* with the osteoblast–osteocyte line. Some characteristics typically associated with osteocytes could therefore have been inherited from osteoblasts and osteochondroprogenitor cells. Demonstration of *SOST* expression by multiple cell types and identification of *SOST* as an evolutionarily conserved key player in bone modeling in fishes expands the relevance of sclerostin to modeling regulation for both paracrine and autocrine signaling. Currently, a wave of new sclerostin-targeting therapies are being explored in an effort to control sclerostin’s antianabolic properties in the treatment of osteoporosis and other skeletal disorders [42–44]. Our findings suggest great potential for the massively speciose clade of fishes, with both osteocytic and anosteocytic members, as a powerful and relevant platform for research in bone physiology, as well as fracture healing and bone therapeutics.

## Materials and methods

### Ethics statement

All in vivo fish experiments were approved by the ethics committee of the Hebrew University of Jerusalem, permit # MD-16-14844-3.

### Fish

Young adult (8–12 months old) medaka (*Oryzias latipes*) and zebrafish (*Danio rerio*) were obtained from commercial fish suppliers (Aquatic Research Organisms, Hampton, NH, USA, and Aquazone, Tzofit, Israel, respectively). The fish were maintained in a controlled environment under a 12 hour:12 hour light/dark cycle at 28°C, in accordance with standard guidelines, and fed appropriate commercial fish feed [45]. For each experiment, the medaka used were laboratory-reared males, hatched in the same month and phenotypically similar. Since zebrafish were obtained from a commercial supplier and thus included both males and females, they exhibited somewhat greater variation in total length. S1 Table and S3 Fig provide details regarding the age and length data of medaka and zebrafish used in the various experiments. Measurements of standard lengths of the fish used in this study can be found in S1 Data.

### Swim-training experimental groups

The investigation reported in this manuscript included 5 separate experimental set-ups, which consisted of the following:

1. Six-week swim-training experiment. This experiment consisted of 4 groups (swim-trained medaka, untrained medaka, swim-trained zebrafish, and untrained zebrafish), each comprising 12 fish. Three fish from each group provided samples for nanoindentation testing, 4 fish from each group were viewed by confocal microscopy to examine fluorochrome distributions, and 5 fish from each group were micro-CT scanned (all 5 from each group were scanned by laboratory micro-CT, and 3 from these 5 from each of the groups were rescanned by synchrotron tomography). The distribution of lengths of fish participating in this experiment is provided in S3 Fig.
2. Five-hour swim-training experiment. This experiment consisted of 2 groups (swim-trained medaka and untrained medaka), each comprising 3 fish. These fish provided samples for the ISH experiment on swimming fish.
3. One-hour swim-training experiment. This experiment consisted of 2 groups, swim-trained medaka (*n* = 5) and untrained medaka (*n* = 7). These fish provided samples for RT-qPCR quantification of *SOST* (swim trained: *n* = 5, untrained: *n* = 7), and Col1a1 (swim trained: *n* = 4, untrained: *n* = 6).
4. Ten-day swim-training experiment. This experiment consisted of 2 groups, swim-trained medaka (*n* = 5) and untrained medaka (*n* = 4). These fish provided samples for RT-qPCR quantification of *SOST* (swim trained: *n* = 5, untrained: *n* = 4), and Col1a1 (swim trained: *n* = 5, untrained: *n* = 4).

### In vivo mechanical loading by swim training

Mechanical loading of fish vertebral columns was achieved by swim training, using a custom-built swim-training device. The device consists of a water pump, a reservoir tank, overflow exits, and 4 training chambers (see videos of untrained and trained fish in S1 Video). The flow rate in each training chamber is separately controlled by a valve and continuously measured by flow meters. Flow through the training chambers is made uniformly semilaminar by the upstream placement of an array of 10-cm–long straws. A mesh screen located downstream, at the end of each chamber, prevents fish from leaving the swim chambers. Prior to the initiation of swim-training experiments, 3 medaka and 3 zebrafish were tested for their critical velocity (swimming velocity at which these fish fatigue), using a previously published protocol [46,47]. The optimal velocity for swim training was then defined as 45% of the critical velocity. During experiments, each of the 4 training chambers contained a different group of fish (control and swim-trained medaka and control and swim-trained zebrafish, *n* = 12 in each group). Temperature and photo period in the swim-training device were kept the same as in the holding tanks. Fish were fed twice daily (before and after training). Untrained (control) fish were kept at a minimal flow rate. The training protocol involved sustained swimming at a constant velocity (26 cm/s for medaka and 33 cm/s for zebrafish, which equals approximately 11 body lengths/s for each species) for 7 hours per day, 5 days per week for 6 weeks.

### Fluorochromes

In order to study the bone-formation process, medaka (12 control and 12 swim-trained fish) and zebrafish (12 control and 12 swim-trained fish) were double labeled by intraperitoneal injections of 2 different fluorochromes. The injections consisted of alizarin red (Sigma Aldrich, St. Louis, MO, USA) on the first day of the experiment (*t* = 0) and of calcein green (Sigma Aldrich) at the end of the experiment (*t* = 6 weeks). All fluorochromes were prepared for injection by modification of the method described previously by Atkins and colleagues [15]. Briefly, alizarin red and calcein green solutions were prepared with 0.2% bicarbonate buffer. Prior to administration, the solutions were sterilized using 0.2 μm Minisart high-flow filters (Sartorius, Göttingen, Germany). Fish were anesthetized with 0.02% tricaine methane-sulfonate (MS-222; Sigma Aldrich) prior to fluorochrome injections. Alizarin red and calcein green were injected into the peritoneal cavity under the guidance of a stereo dissection microscope at a dose of 50 mg/kg and 0.5 mg/kg, respectively, using a microsyringe (Microliter syringe; Hamilton, Reno, NV, USA). After injections, fish were allowed to recuperate in an isolated tank containing clean water. All fish recovered uneventfully from all injections, except for 1 medaka that was excluded from the experiment.

### Samples

At the end of the swim-training experiment, fish were removed from the training chambers and killed with an overdose of tricaine methane-sulfonate (MS-222; Sigma Aldrich, USA). The caudal part of the vertebral columns was gently dissected and manually cleaned of soft tissue using a stereo dissection microscope. Caudal vertebrae are numbered from caudal to cranial such that the first caudal vertebra is the most caudal. We used the fourth caudal vertebra (shown in Fig 1) in all experiments, except for ISH and RT-qPCR experiments, as detailed below. The harvested tissues were further processed as described below.

### Confocal microscopy

The fourth caudal vertebrae of swim-trained and control medaka and zebrafish were imaged by confocal microscopy (Leica SP8 microscope, Leica, Wetzlar Germany) to study the precipitation patterns of the injected fluorochromes. The excitation/emission wavelengths used to observe fluorochromes were 543/580–670 nm and 488/500–535 nm for alizarin red and calcein green, respectively. Z-stack images of a comparable caudal vertebra were viewed using ImageJ/FIJI (FIJI v. 1.51r, NIH, Bethesda, MD, USA) in the Maximum Intensity Projection (MIP) mode in order to evaluate bone formation during the experiment.

### Laboratory-source–based microcomputed tomography

The fourth caudal vertebrae of trained and untrained fish (dissected from fluorochrome-stained medaka and zebrafish) were scanned with a desktop micro-CT scanner (1172 scanner, SkyScan; Bruker, Kontich, Belgium) in order to obtain their 3D morphology. The X-ray source was set at 50 kV and 200 μA. 4,000 projections were acquired for each scan over an angular range of 360 degrees. The scans had an isotropic voxel size of 2μm, and exposer time was 3.5 seconds. All scans were performed with a 0.5 mm aluminum filter, in order to decrease beam-hardening effects. Scans were reconstructed using commercial software (NRecon Skyscan software, SkyScan; Bruker Kontich, Belgium). Reconstructed scans were volume rendered (Amira software v.6.3, FEI, Hillsboro, OR, USA) to visualize the 3D morphology of the selected vertebra or segmented and meshed to create the geometry for an FE model (described below).

In order to study the 3D morphology of the paravertebral musculature, the caudal half of a medaka (including soft tissues) was gently cleaned from scales and skin, using a stereo dissection microscope. The tissue was fixed overnight in 4% PFA and dehydrated in increasing concentrations of ethanol (25%, 50%, and 70%). In order to improve the contrast of the soft tissues, the sample was stained with 0.3% phosphotungstic acid solution (PTA; Sigma Aldrich) in 70% ethanol for 6.5 days. After PTA staining, the sample was washed in 70% ethanol to remove residues of PTA and scanned using a micro-CT scanner (XRadia MICRO XCT-400; Zeiss, Thornwood, NY, USA). The X-ray source was set at 40 kV and 200 μA. In total, 1,200 projections were acquired over an angular range of 180 degrees. The scans were made with an isotropic voxel size of 2.57μm and an exposure time of 3 seconds. Scans were reconstructed using XRadia software, using a filtered back-projection algorithm. The reconstructed scan was then volume rendered using Amira software v.6.3 (FEI) in order to visualize the musculature–bone inter-relationships.

### Synchrotron-based microcomputed tomography

For higher-resolution tomography and visualization of submicron features, the fourth caudal vertebrae of trained and untrained medaka and zebrafish were scanned using synchrotron-based microtomography (SμCT). Scans were performed at beamline ID19 of the European Synchrotron Radiation Facility (ESRF, Grenoble, France). The samples were scanned using X-ray photon energy of 34 keV. A total of 4,000 radiographic projection images were recorded over 180° with an exposure time of 0.2 seconds and an effective pixel size of 650 nm. Propagation-based X-ray phase-contrast enhancement was induced using a sample-detector distance of 88 mm. ESRF in-house code was used to reconstruct the data, where voids and interfaces were enhanced by means of Paganin-based filtering [48]. The reconstructed scans were viewed with Amira software v.6.3 (FEI). For 3D visualization of osteocytic lacunae, mineralized tissue and voids were separately segmented and rendered with different colors. Both zebrafish and medaka vertebrae underwent the exact same semiautomated segmentation.

### Nanoindentation

In order to determine the mechanical properties of the bone material, the fourth caudal vertebrae of 6 medaka (3 controls and 3 swim-trained fish) and 6 zebrafish (3 controls and 3 swim-trained fish) were dehydrated with increasing concentrations of ethanol and embedded for 8 hours in polymethylmethacrylate (PMMA), which was polymerized in an oven at 60°C. The embedded vertebral columns were cut for indentation as ca. 0.5-mm–thick transverse and longitudinal sections using an Isomet slow-speed water-cooled diamond-blade saw (Buehler, Lake Bluff, IL, USA). The slices were ground with 3 μm and 1 μm grit SiC lms (Buehler), then polished with nap cloth soaked with diamond suspension (0.25 μm; Struers, Cleveland, OH, USA) or alumina suspension (0.25 μm; Buehler). The polished sections were nanoindented using a scanning nanoindenter (Ubi 1, Hysitron, Billerica, MA, USA) with a Berkovic indenter tip [49]. An optical microscope, aligned with the nanoindenter tip, was used to locate regions of interest on the evenly polished bone surface. The following load function was used: maximum load of 2.5 mN, loading at 0.5 mN s^−1^, holding at maximum force for 60 seconds, and unloading to 0.5 mN at a rate of 0.2 mN s^−1^, followed by a second holding time of 20 seconds, and finally unloading to 0 mN at a rate of 0.1 mN s^−1^. Young's modulus of the material was calculated using the Oliver–Pharr method, based on the slope of the unloading curve in the region between 20% and 95% of the maximum load [50]. Following indentation, the samples were coated with gold and examined with a scanning electron microscope (JCM 6000 benchtop SEM; Jeol, Peabody, MA, USA) to verify the quality and position of the indentations. Unacceptable indents (e.g., indents that were in the embedding material) were discarded. Original raw data for all indentation-determined Young’s moduli values are presented in S1 Data.

### FEA

The reconstructed micro-CT scan of a representative caudal vertebra of medaka was imported into Amira software v.6.3 (FEI). All 2D slices of the scan were semiautomatically segmented by selecting an appropriate threshold and manually correcting when necessary, and a 3D model of the vertebra was created. The same software package was used to mesh the model with tetrahedral elements, resulting in 386,289 10-node tetrahedral elements. The meshed model was then exported into an FEA software package (Patran 2017r1; MSC, USA). Fig 3 shows detailed features of the FE model. The bone material in the model was assumed isotropic and linearly elastic. Poisson’s ratio was taken to be 0.3. Young’s moduli were assigned based on nanoindention results and attenuation values in the micro-CT scan. Specifically, the attenuation values of the micro-CT scan were divided into 10 equally spaced bins, and a custom-written MATLAB code (MATLAB R2016b, The MathWorks, Natick, MA, USA) identified each tetrahedral element with the corresponding attenuation value of the voxel in its position. The range of Young's moduli obtained by nanoindentation of several vertebrae was between 6 GPa and 28 GPa, although the majority of elements had moduli in the range of 18 GPa to 22 GPa (see S2 Fig). The range of values was similarly divided into 10 bins, and each of the 10 bins of attenuation values was assigned a corresponding Young's modulus value. As a result, each element of the model received one of 10 Young's modulus values, distributed according to the level of mineralization in different regions of the vertebra.

The main challenge in creating a valid FE model of a physiologically loaded fish vertebra is to apply physiologically reasonable forces (magnitudes and directions). Such data are not available in the literature, and therefore the contrast-enhanced scan of the caudal vertebrae described above (see muscles and vertebrae in S1 Fig) was studied to determine the approximate size, fiber orientation, and regions of insertion of the paravertebral muscles attached to the caudal vertebra selected for the FE model. These data provided approximate muscle force application regions and muscle force directions. The force magnitudes used in the model were based on scaling muscle forces described in previous publications [51,52], such that the ratios of muscle forces reflected their relative cross-sections. It should be noted that since the objective here was to find the relative distribution of strains in the vertebra and since the model is linearly elastic, only the relative magnitudes of the muscle forces are needed.

### Histology and ISH

For histology and ISH, the caudal regions (caudal vertebrae 1–10) of medaka and zebrafish (after skin and scale removal) were fixed in 4% paraformaldehyde (PFA/PBS) for 24 hours at 4°C while being shaken gently. After fixation, the tissues were decalcified for 24 hours in 0.5 M EDTA (pH 7.4), dehydrated in increasing concentrations of ethanol, and imbedded in paraffin. The embedded tissues were cut into 7-μm–thick sagittal sections, which were mounted onto glass slides. HE staining was performed following standard protocols. The RNA probes for ISH were prepared by in vitro transcription of the reverse transcriptase cDNA fragments by using T7 RNA polymerase. Single nonfluorescent ISH was performed using a digoxigenin (DIG)-labeled probe for medaka *SOST*. Double fluorescence ISH (FISH) was performed using fluorescein- and DIG-labeled probes. ISH and FISH were performed following the protocol described by Shwartz and Zelzer [53]. After hybridization, slides were washed, quenched, and blocked. Hybridization probes for single nonfluorescent ISH were detected by incubation with alkaline-phosphatase–conjugated anti-digoxigenin antibody (anti-DIG-AP; Roche, Basel, Switzerland). Hybridization probes for double FISH were detected by incubation with peroxidase-conjugated anti-digoxigenin antibody (anti-DIG-POD, 1:300; Roche) and peroxidase-conjugated anti-fluorescein antibody (anti-fluorescein-POD, 1:200; Roche) followed by Cy2- and Cy3-tyramide-labeled fluorescent dyes according to the instructions of the TSA Plus Fluorescent Systems Kit (Perkin Elmer, Waltham, MA, USA). All primers that were used to generate the probes are listed in S2 Table, some of them based on previous publications [54–57]. ISH results for 2 vertebral regions in medaka and zebrafish are shown in black and white in S4 Fig and S5 Fig, respectively.

### RT-qPCR

For gene expression analyses, 2 separate swim-training experiments were conducted. In order to evaluate the immediate response to swim training, medaka were trained for 1 hour, allowed to rest for 3 hours, and then killed. To evaluate the late response to swim training, medaka were trained for 10 days, allowed to rest overnight, and then killed. *SOST* and *Col1a1* expression levels were analyzed to evaluate the effect of mechanical loading on *SOST* expression and osteoblastic activity. Caudal vertebrae 1–10 of 1-hour swim-trained medaka, 10-day swim-trained medaka, and untrained medaka (control) were separately homogenized in TRI-reagent (Sigma Aldrich) using a tissue homogenizer. Total RNA was isolated from the tissues according to manufacturer’s instructions. RNA quality and concentration were verified by NanoDrop spectrophotometry. One μg of RNA was reverse transcribed to cDNA with a qScript cDNA Synthesis Kit (Quanta Biosciences, Gaithersburg, MD, USA). Transcript levels of *SOST* and *Col1a1* were analyzed using PerfeCTa SYBR Green SuperMix (Quanta Biosciences) on a StepOne Real-Time PCR system (Applied Biosystems, Foster City, CA, USA) and normalized to housekeeping gene (*RPL-7)* levels. All primers used for gene expression analysis are listed in S2 Table. Relative expression was calculated using the delta-delta Ct standardization method. Statistical analysis of the results employed Student *t* test (two-sided). *P*-values < 0.05 were considered to be statistically significant.

### Morpholino-mediated *SOST* knockdown

Custom splice-site–targeted MO oligonucleotides for the *SOST* gene (5′- AAAAGGACACTTACTATATGAAACTGT-3′) and standard control oligonucleotides were purchased from Gene-Tools (Philomath, OR, USA). The custom MO was designed against the donor splice site to block *SOST* gene pre-mRNA splicing in adult medaka. Two μl of the MO (0.5 mM diluted with PBS [pH 7.4]) were injected to the ventral side of the fourth caudal vertebra using a microsyringe (Microliter syringe, Hamilton) under the guidance of a stereo dissecting microscope. The semitransparent body wall of medaka allowed clear visualization of the vertebra and the site of injection. Injections of *SOST*-targeted and standard control MOs were made every 3 days for 2.5 weeks in 5 medaka and 4 medaka, respectively. For evaluation of the effect of *SOST* knockdown on bone formation, we injected alizarin red prior to MO injections and calcein green at the end of the experiment. Fluorochrome staining was imaged by confocal microscopy (as described above). In order to validate the efficiency of the splice-blocking *SOST* MO, both the ventral and dorsal sides of the caudal vertebral column of 4 control medaka and 10 MO-injected medaka were injected with 1.5 μl standard control or *SOST* MO, respectively. One to 4 hours after a single injection, fish were killed, and their caudal vertebral columns were harvested. Each 2 vertebral columns of fish from the same group were homogenized together in TRI-reagent (Sigma Aldrich, USA) using a tissue homogenizer. Total RNA was isolated from the tissues according to manufacturer’s instructions. RNA quality and concentration were verified by NanoDrop spectrophotometry. One μg of RNA was reverse transcribed to cDNA with a qScript cDNA Synthesis Kit (Quanta Biosciences). Primers (listed in S2 Table) for reverse transcription PCR (RT-PCR) were designed to detect the intron insertion of the morpholino-affected transcripts. RT-PCR products were separated by electrophoresis in 2% agarose gels and visualized by ethidium bromide staining. The splice modification was verified by RT-PCR reaction, using an E1 and an i1, which showed insertion of the intronic sequence into to the morphant transcripts (see S6 Fig). All primers used here were specific to *SOST* on chromosome 19. Two other paralogues of *SOST*—on chromosomes 16 and 11, respectively—were not used in this study, though we do not exclude the possibility that the 2 additional paralogues may also participate in the regulation of modeling.

## Supporting information

S1 Video Swim training. (a) Medaka swim trained against a water current with tightly controlled (26 cm/s) velocity. Note that fish are swimming against the current, station-holding, and not turning. (b) Untrained (control) medaka swimming in a similar-sized chamber as the swim-training chamber, with minimal speed water flow (only sufficient for maintaining fresh water). Fish are swimming slowly in all directions.
(MP4)

S1 Fig Musculature and bone in the caudal region of medaka. Caudal is to the right in all images. (a) Sagittal section of a 3D reconstruction of contrast-enhanced (PTA) tomography showing the dense musculature surrounding the caudal vertebral column of medaka. One vertebra is segmented in white to show the full 3D muscle–vertebral relationship. The caudal faces of the vertebral C, NS, and HS are indicated. Neural arch muscular attachments are marked by white arrows. (b) Medial view of a sagittal section of medaka caudal musculature, with vertebrae digitally removed. The paravertebral musculature is complex and robust and occupies a large portion of the caudal part of the fish body, attaching mainly to the NS and HS and arches. Muscle fiber orientations and sites of attachment are clearly visible: intervertebral joints occupy the serial, diamond-shaped gaps in the musculature, and hemal arch muscular attachments are marked by white arrows. C, centrum; HS, hemal spine; NS, neural spine; PTA, phosphotungstic acid.
(TIF)

S2 Fig Young’s moduli derived from nanoindentation of the vertebral body of swim-trained and untrained medaka and zebrafish. *N* = 30–65 indents from 3 fish of each group. Quantitative data were compared between groups by the nonparametric Mann–Whitney test, with the level of significance set at *P* ≤ 0.05. All original measurements of Young’s moduli determined by nanoindentation are presented in S1 Data.
(TIF)

S3 Fig Distribution of fish lengths (mm) in the first swimming experiment. No significant difference was found between untrained and trained fish of either species. There was a significant difference between medaka and zebrafish average lengths; as a result, higher swimming speeds were used for zebrafish in swimming experiments.
(TIF)

S4 Fig ISH results for 2 vertebral regions in medaka. Top row: NS and adjacent fin R; bottom row: intervertebral J. Each row comprises (from left to right) a multichannel RGB double fluorescent ISH image, followed by isolated single-channel images (DAPI, col1, col2, respectively) and a SOST ISH image. The figure provides a deconstruction of the multichannel ISH images in Fig 4. Note SOST-positive Cbs, Chs, and Obs and the lack of osteocytes within the NS. Refer to text for further results. Cb, chordoblast; Ch, chondrocyte; ISH, in situ hybridization; J, joint; NS, neural spine; Ob, osteoblast; R, radial; RGB, red-green-blue.
(TIF)

S5 Fig ISH results for 2 vertebral regions in zebrafish. Top row: NS; bottom row: intervertebral J. Each row comprises (from left to right) a multichannel RGB double fluorescent ISH image, followed by isolated single-channel images: DAPI, collagen (*col1a1* = top row, *col2a1* = bottom row), SOST. The figure provides a deconstruction of the multichannel ISH images in Fig 5; the bottom row, however, is a different section from the one shown in Fig 5j. Note SOST-positive Cbs and Obs and the presence of osteocytes (white arrowheads) within the bone, which express little to no SOST. Refer to text for further results. Cb, chordoblast; *Col1a1*, collagen type I alpha 1; *Col2a1*, collagen type II alpha 1; ISH, in situ hybridization; J, joint; NS, neural spine; Ob, osteoblast; RGB, red-green-blue.
(TIF)

S6 Fig Sequencing results of the MO-injected transcript compared with the medaka genome. The primer sequences used are shown in red letters. The exon parts are highlighted in blue, and the intron parts are highlighted in yellow. A stop codon present on the intron that was retained is highlighted in red. The sequencing shows that a significant portion of the intron was inserted into the transcript. This insertion very likely disrupted the proper translation of the protein, especially since the presence of a stop codon probably completely eliminated the translation of the second exon. MO, vivo-morpholino.
(DOCX)

S1 Table List of ages of medaka used in different experiments. All original measurements of the total length of the fish participating in the different experimental groups are presented in S1 Data.
(DOCX)

S2 Table List of primers used. (DOCX)

S1 Data Underlying numeric data used in this work. (XLSX)

## Abbreviations

anti-DIG-AP: alkaline-phosphatase–conjugated anti-digoxigenin antibody
anti-DIG-POD: peroxidase-conjugated anti-digoxigenin antibody
anti-fluorescein-POD: peroxidase-conjugated anti-fluorescein antibody
Col1a1: collagen type I alpha 1
Col2a1: collagen type II alpha 1
DIG: digoxigenin
ESRF: European Synchotron Radiation Facility
E1: exon-spanning forward primer
FE: finite element
FEA: finite element analysis
FISH: fluorescence in situ hybridization
HE: hematoxylin–eosin
HS: hemal spine
ISH: in situ hybridization
IVS: intervertebral space
i1: intron-spanning reverse primer
J: joint
MIP: Maximum Intensity Projection
MO: vivo-morpholino
NS: neural spine
O: osteocytic lacunae
PCR: polymerase chain reaction
PFA: paraformaldehyde
PMMA: polymethylmethacrylate
PTA: phosphotungstic acid
R: radial
RT-PCR: reverse transcription polymerase chain reaction
RT-qPCR: real-time quantitative polymerase chain reaction
SEM: scanning electron microscope
SμCT: synchotron-based microtomography
VB: vertebral bone
