# Supplementary figures and images for: A novel nonosteocytic regulatory mechanism of bone modeling
Source: PLoS Biol. 2019 Feb 1;17(2):e3000140. doi: 10.1371/journal.pbio.3000140 (PMC6373971; doi:10.1371/journal.pbio.3000140)

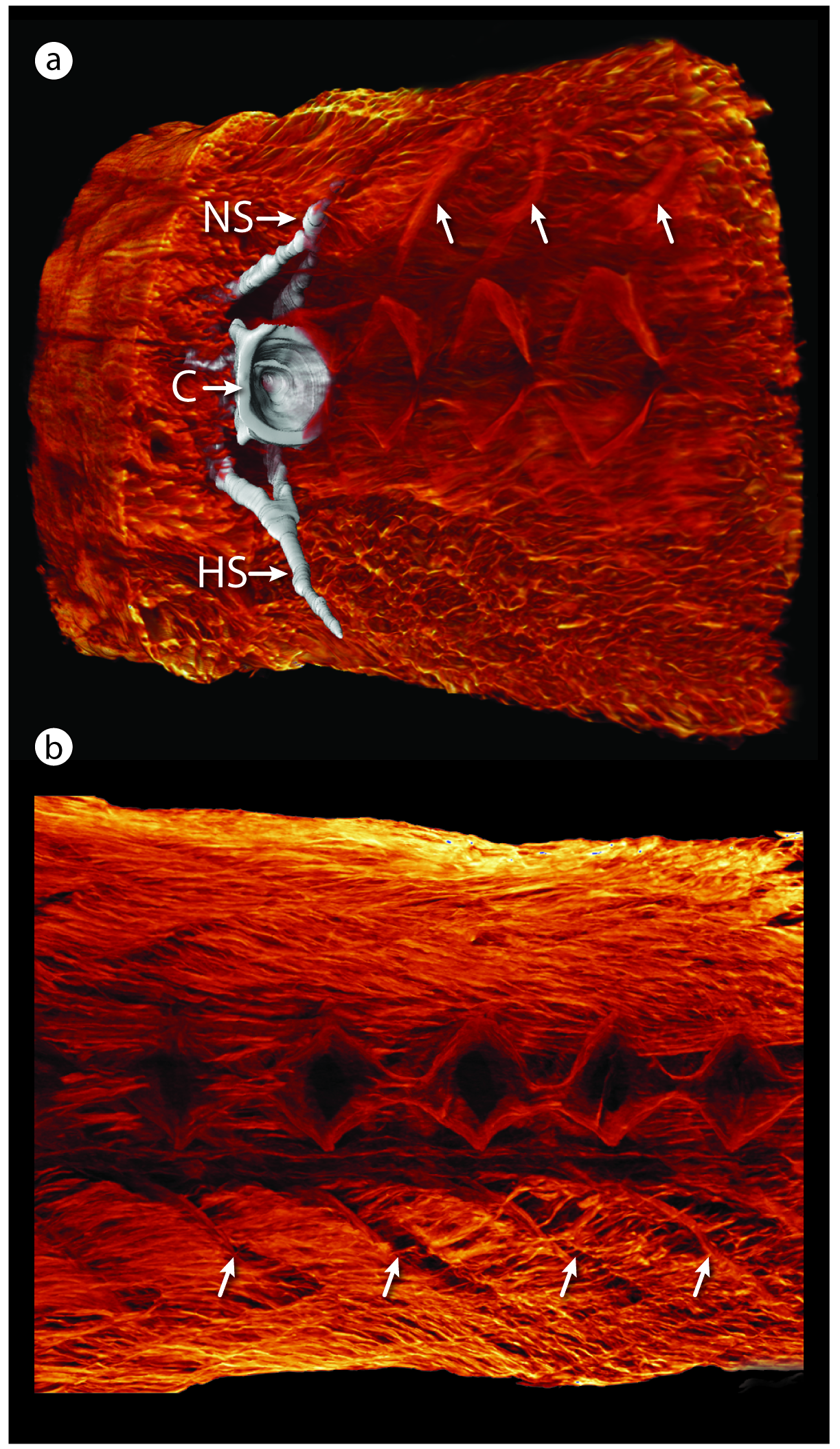

Supplement: S1 Fig — Caudal is to the right in all images. (a) Sagittal section of a 3D reconstruction of contrast-enhanced (PTA) tomography showing the dense musculature surrounding the caudal vertebral column of medaka. One vertebra is segmented in white to show the full 3D muscle–vertebral relationship. The caudal faces of the vertebral C, NS, and HS are indicated. Neural arch muscular attachments are marked by white arrows. (b) Medial view of a sagittal section of medaka caudal musculature, with vertebrae digitally removed. The paravertebral musculature is complex and robust and occupies a large portion of the caudal part of the fish body, attaching mainly to the NS and HS and arches. Muscle fiber orientations and sites of attachment are clearly visible: intervertebral joints occupy the serial, diamond-shaped gaps in the musculature, and hemal arch muscular attachments are marked by white arrows. C, centrum; HS, hemal spine; NS, neural spine; PTA, phosphotungstic acid. (TIF) [file pbio.3000140.s002.tif]

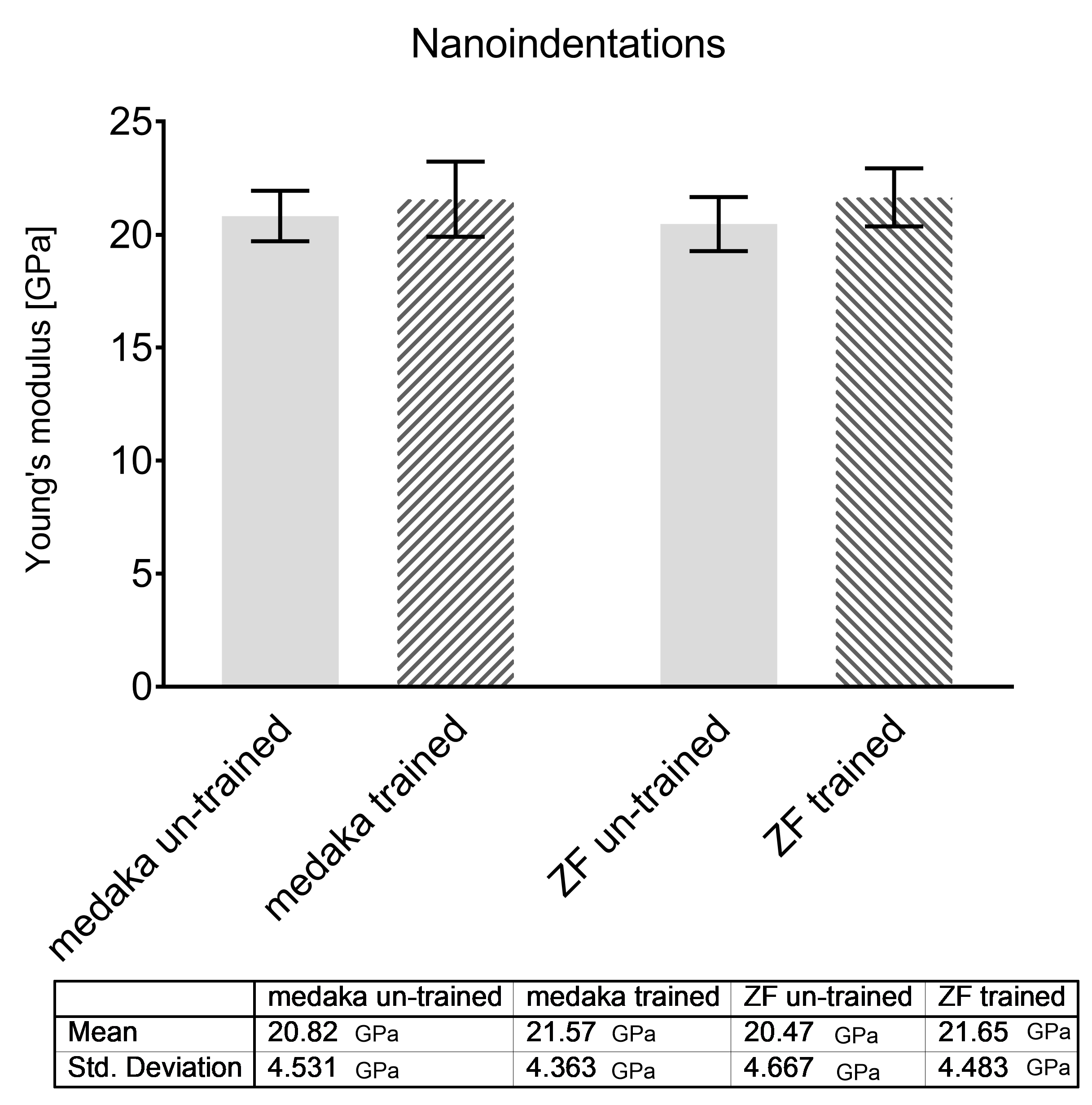

Supplement: S2 Fig — N = 30–65 indents from 3 fish of each group. Quantitative data were compared between groups by the nonparametric Mann–Whitney test, with the level of significance set at P ≤ 0.05. All original measurements of Young’s moduli determined by nanoindentation are presented in S1 Data. (TIF) [file pbio.3000140.s003.tif]

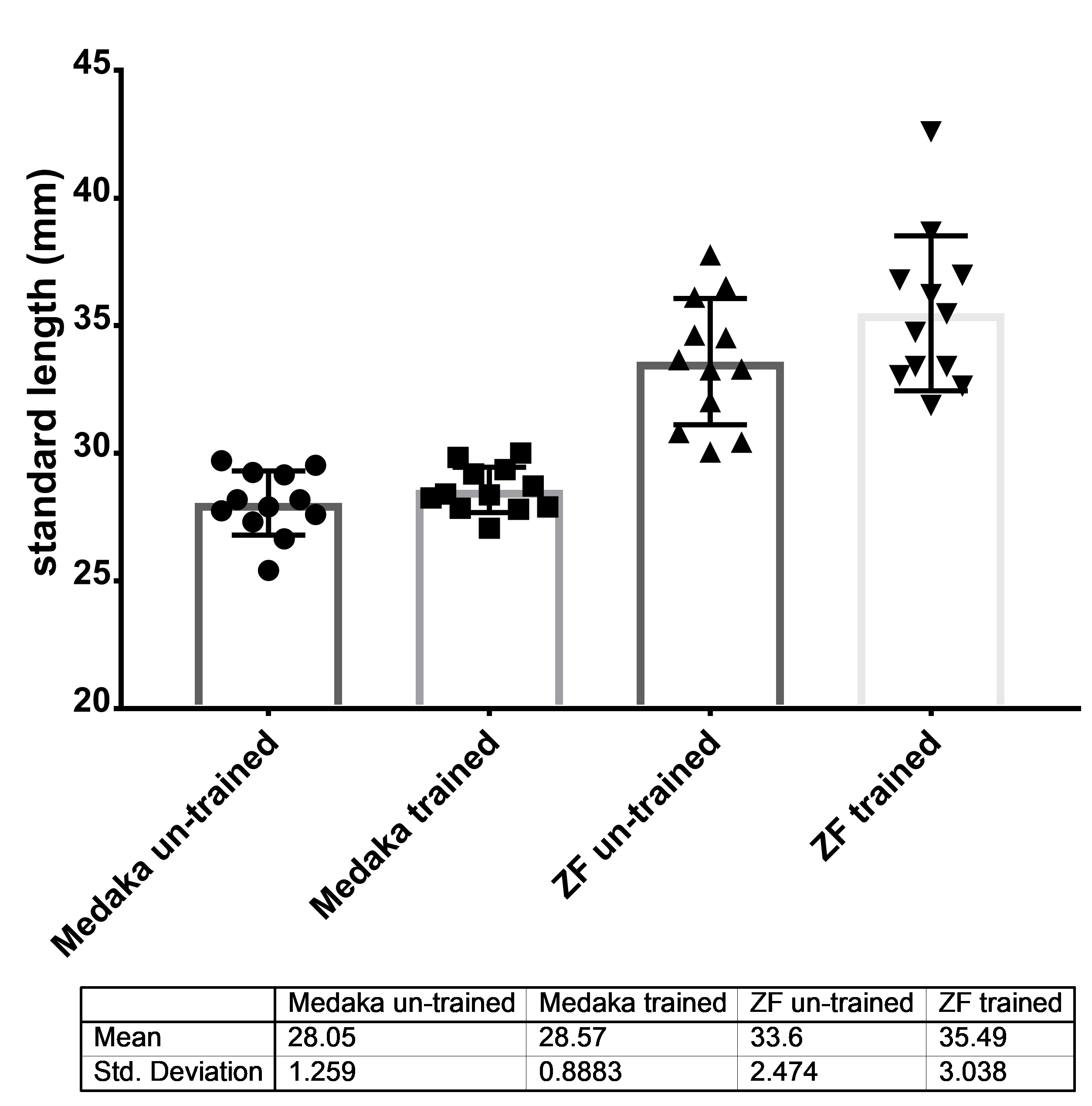

Supplement: S3 Fig — No significant difference was found between untrained and trained fish of either species. There was a significant difference between medaka and zebrafish average lengths; as a result, higher swimming speeds were used for zebrafish in swimming experiments. (TIF) [file pbio.3000140.s004.tif]

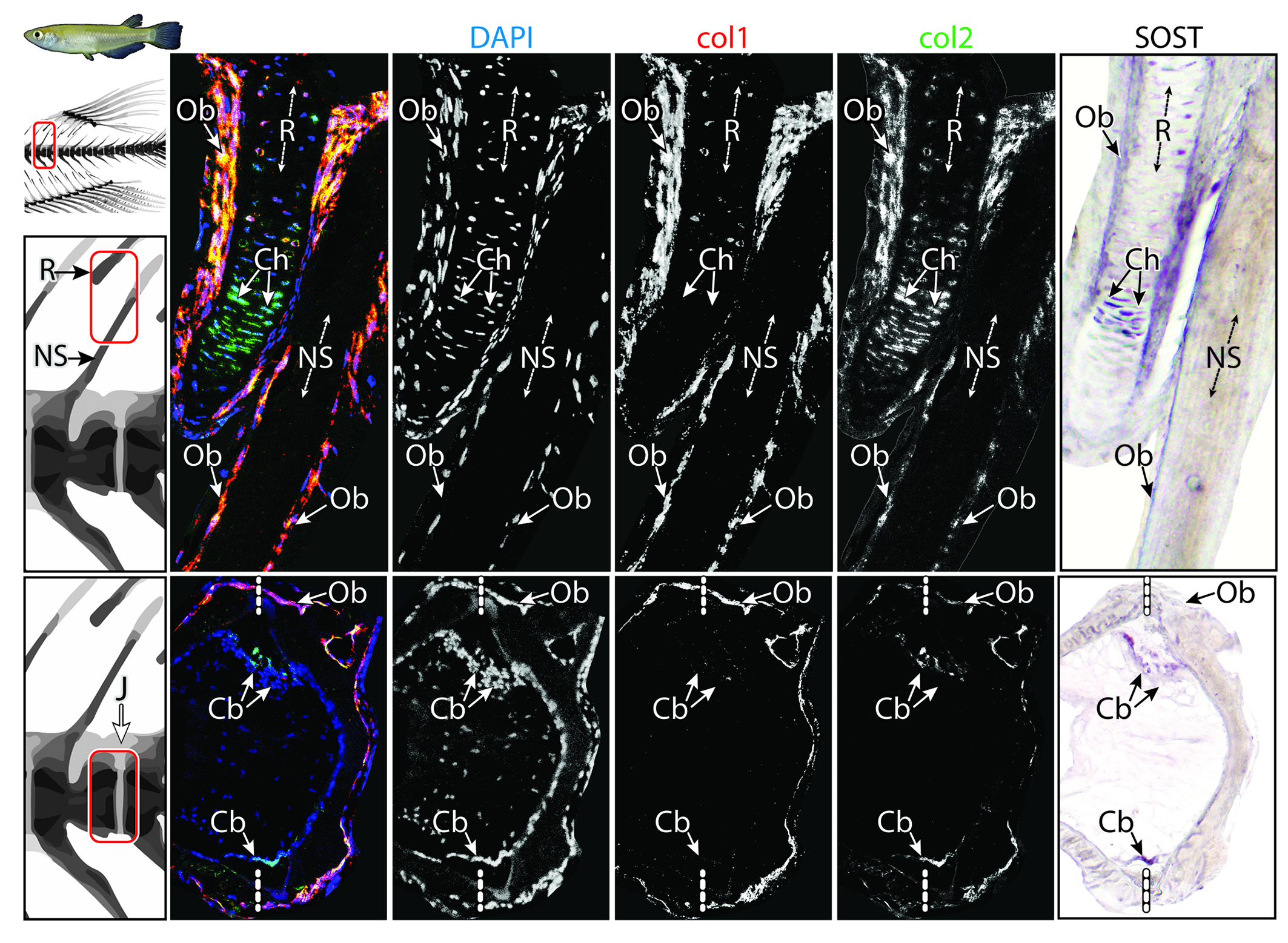

Supplement: S4 Fig — Top row: NS and adjacent fin R; bottom row: intervertebral J. Each row comprises (from left to right) a multichannel RGB double fluorescent ISH image, followed by isolated single-channel images (DAPI, col1, col2, respectively) and a SOST ISH image. The figure provides a deconstruction of the multichannel ISH images in Fig 4. Note SOST-positive Cbs, Chs, and Obs and the lack of osteocytes within the NS. Refer to text for further results. Cb, chordoblast; Ch, chondrocyte; ISH, in situ hybridization; J, joint; NS, neural spine; Ob, osteoblast; R, radial; RGB, red-green-blue. (TIF) [file pbio.3000140.s005.tif]

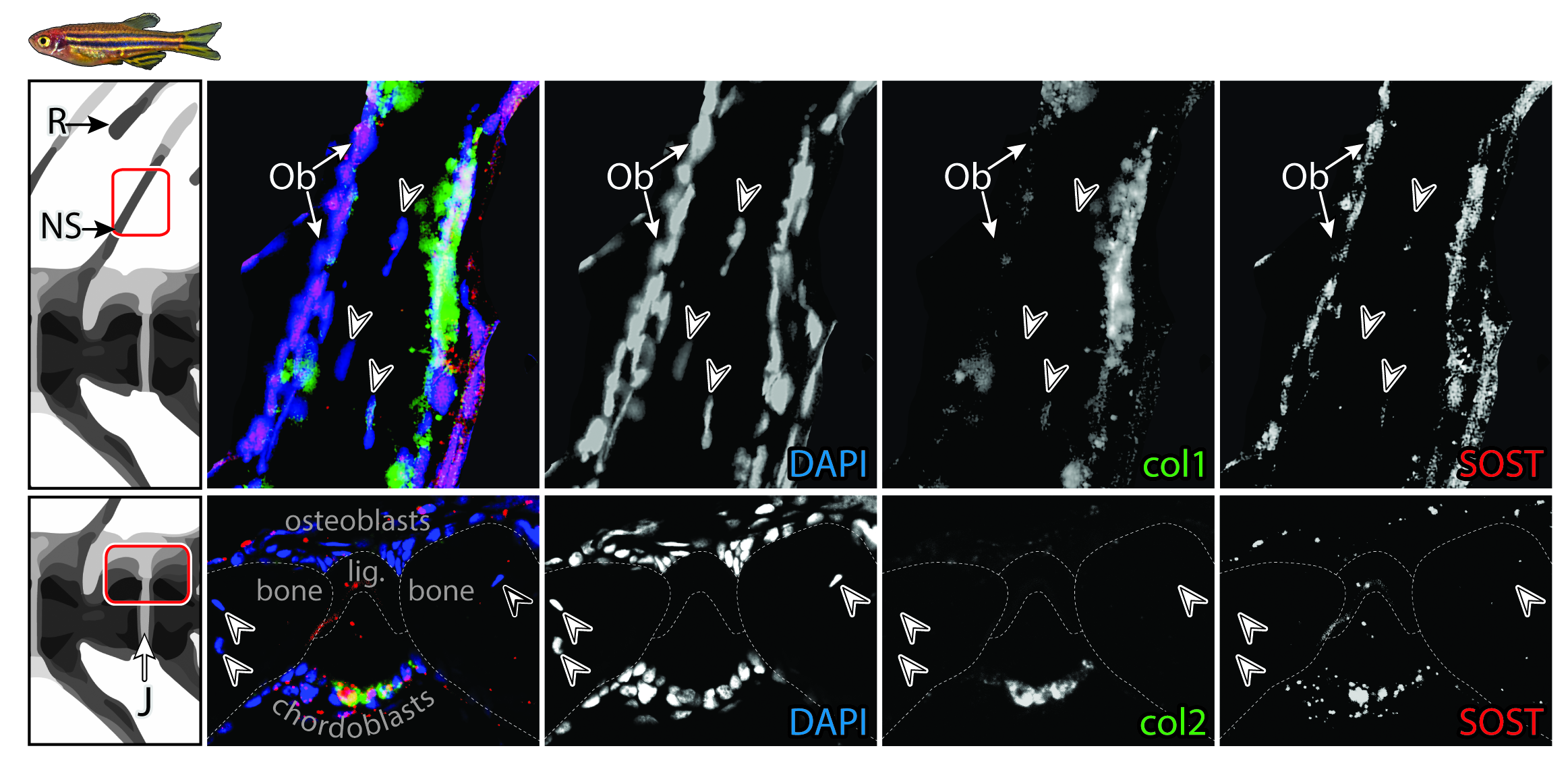

Supplement: S5 Fig — Top row: NS; bottom row: intervertebral J. Each row comprises (from left to right) a multichannel RGB double fluorescent ISH image, followed by isolated single-channel images: DAPI, collagen (col1a1 = top row, col2a1 = bottom row), SOST. The figure provides a deconstruction of the multichannel ISH images in Fig 5; the bottom row, however, is a different section from the one shown in Fig 5j. Note SOST-positive Cbs and Obs and the presence of osteocytes (white arrowheads) within the bone, which express little to no SOST. Refer to text for further results. Cb, chordoblast; Col1a1, collagen type I alpha 1; Col2a1, collagen type II alpha 1; ISH, in situ hybridization; J, joint; NS, neural spine; Ob, osteoblast; RGB, red-green-blue. (TIF) [file pbio.3000140.s006.tif]
