# Supplementary material for: A novel nonosteocytic regulatory mechanism of bone modeling
Source: PLoS Biol. 2019 Feb 1;17(2):e3000140. doi: 10.1371/journal.pbio.3000140 (PMC6373971; doi:10.1371/journal.pbio.3000140)
Supplement: S6 Fig — The primer sequences used are shown in red letters. The exon parts are highlighted in blue, and the intron parts are highlighted in yellow. A stop codon present on the intron that was retained is highlighted in red. The sequencing shows that a significant portion of the intron was inserted into the transcript. This insertion very likely disrupted the proper translation of the protein, especially since the presence of a stop codon probably completely eliminated the translation of the second exon. MO, vivo-morpholino. (DOCX) [file pbio.3000140.s007.docx]

Sequencing compared to Medaka chr19: 16317859-16318003 (oryLat2, UCSC genome browser)

MO 00000001 aacactttgaataacagggcgaaaaacggtggaaggactgcaaacacagt 00000050

>>>>>>>> |||||||||||||||||||||||||||||||||||||||||||||||||| >>>>>>>>

sost 16317859 aacactttgaataacagggcgaaaaacggtggaaggactgcaaacacagt 16317908

MO 00000051 ttcatatagtaagtgtccttttaatatttaaatattacatcaattaccta 00000100

>>>>>>>> |||||||||||||||||||||||||||||||||||||||||||||||||| >>>>>>>>

sost 16317909 ttcatatagtaagtgtccttttaatatttaaatattacatcaattaccta 16317958

MO 00000101 tttgggcaaactttgtagaaaaatgtggtgaagataagcacatgg 00000145

>>>>>>>> ||||||||||||||||||||||||||||||||||||||||||||| >>>>>>>>

sost 16317959 tttgggcaaactttgtagaaaaatgtggtgaagataagcacatgg 16318003
