# Supplementary material for: A novel nonosteocytic regulatory mechanism of bone modeling
Source: PLoS Biol. 2019 Feb 1;17(2):e3000140. doi: 10.1371/journal.pbio.3000140 (PMC6373971; doi:10.1371/journal.pbio.3000140)
Supplement: S1 Table — All original measurements of the total length of the fish participating in the different experimental groups are presented in S1 Data. (DOCX) [file pbio.3000140.s008.docx]

| **In situ hybridization primers** | | | |  |  |
| --- | --- | --- | --- | --- | --- |
|  |  |  |  |  |  |
| organism | gene | NCBI gene ID | Forward | Reverse | previous publications |
| medaka | sost | [XM_004080635.3](https://www.ncbi.nlm.nih.gov/entrez/viewer.fcgi?db=nucleotide&id=1174668235) | TGCAAACACAGTTTCATATAGTGCT | GTGCCAGCTGAGGTTTAGGT |  |
| medaka | col2a1 | [XM_011477401.2](https://www.ncbi.nlm.nih.gov/entrez/viewer.fcgi?db=nucleotide&id=1174692701) | GAATCAGCAAAGTACCAAAG | ACCGGCCTGAATGCCTCTT | Dong et al. 2012 ^54^ |
| medaka | col1a1 | [NM_001122918.2](https://www.ncbi.nlm.nih.gov/entrez/viewer.fcgi?db=nucleotide&id=1174098373) | TGTTCCGTGCTGATGATGCT | ATGTACCGGTGTGTGACGTG |  |
| zebrafish | sost | [XM_001340647.5](https://www.ncbi.nlm.nih.gov/entrez/viewer.fcgi?db=nucleotide&id=1207190415) | ACACACGGACTTATGGAGCC | TGAATTGCTGTTGATGGACGG |  |
| zebrafish | col2a1a | [XM_005166863.4](https://www.ncbi.nlm.nih.gov/entrez/viewer.fcgi?db=nucleotide&id=1207182926) | GCAAAGGGACAGAAAGGAGAACCA | CACCATCACTTCCGGGTTTTCCA | Shwartz et al. 2012 ^55^ |
| zebrafish | col1a1a | [NM_199214.1](https://www.ncbi.nlm.nih.gov/entrez/viewer.fcgi?db=nucleotide&id=56790314) | GTGATCTGCGAAGACACAAGCGA | AATCCTCTGTGTCCCTTGATGCCT |  |
|  |  |  |  |  |  |
|  |  |  |  |  |  |
| **qRT-PCR primers** | | |  |  |  |
|  |  |  |  |  |  |
| organism | gene | NCBI gene ID | Forward | Reverse |  |
| medaka | sost | [XM_004080635.3](https://www.ncbi.nlm.nih.gov/entrez/viewer.fcgi?db=nucleotide&id=1174668235) | GCGAAAAACGGTGGAAGGAC | TCGGTAATGTAGCGGGTGGA |  |
| medaka | col1a1 | [NM_001122918.1](https://www.ncbi.nlm.nih.gov/nucleotide/171544946?report=genbank&log$=nuclalign&blast_rank=2&RID=1EP8T5K6015) | GCTCTTTGCCAGAGGATGTC | TCATTGGAACCTTGGAGGAG | Watson et al. 2017^56^ |
| medaka | rpl7* | [NM_001104870.1](https://www.ncbi.nlm.nih.gov/entrez/viewer.fcgi?db=nucleotide&id=157278476) | CGCCAGATCTTCAACGGTGTAT | AGGCTCAGCAATCCTCAGCAT | Zhang et al. 2006 ^57^ |
|  |  |  |  |  |  |
| *housekeeping gene | |  |  |  |  |
|  |  |  |  |  |  |
| **splice blocking validation (for morpholino experiment)** | | | |  |  |
|  |  |  |  |  |  |
| organism | gene |  | Forward | Reverse (on intron) |  |
| medaka | sost | validation pair 1 | AAC ACT TTG AAT AAC AGG GCG | CCA TGT GCT TAT CTT CAC CAC |  |
| medaka | sost | validation pair 2 | GCG AAA AAC GGT GGA AGG AC | ACC CAT GTG CTT ATC TTC ACC A |  |

**SI Table 1 – list of primers used.**
