# Supplementary material for: A novel nonosteocytic regulatory mechanism of bone modeling
Source: PLoS Biol. 2019 Feb 1;17(2):e3000140. doi: 10.1371/journal.pbio.3000140 (PMC6373971; doi:10.1371/journal.pbio.3000140)
Supplement: S2 Table — (DOCX) [file pbio.3000140.s009.docx]

|  | experiment date | hatched | Age (months) |
| --- | --- | --- | --- |
| Swim-training experiment | Dec- 2015 | Feb-2015 | ±10 |
| Morpholino experiment | Nov-2016 | Feb-2016 | ±9 |
| in situ hybridization experiment | Feb-2017 | Feb-2016 | ±12 |
| real time PCR experiment | Sep-2017 | Feb-2017 | ±8 |
